# Characterization of the UDP-glycosyltransferase UGT72 Family in Poplar and Identification of Genes Involved in the Glycosylation of Monolignols

**DOI:** 10.3390/ijms21145018

**Published:** 2020-07-16

**Authors:** Nathanael Speeckaert, Nassirou Mahamadou Adamou, Hadjara Amadou Hassane, Fabien Baldacci-Cresp, Adeline Mol, Geert Goeminne, Wout Boerjan, Pierre Duez, Simon Hawkins, Godfrey Neutelings, Thomas Hoffmann, Wilfried Schwab, Mondher El Jaziri, Marc Behr, Marie Baucher

**Affiliations:** 1 Laboratoire de Biotechnologie Végétale, Université libre de Bruxelles (ULB), Rue des Professeurs Jeener et Brachet 12, 6041 Gosselies, Belgium; naspeeck@ulb.ac.be (N.S.); nassirou.mahamadou.adamou@ulb.ac.be (N.M.A.); hadjara.amadou.hassane@ulb.ac.be (H.A.H.); fabien.baldaccicresp@gmail.com (F.B.-C.); adelimol@ulb.ac.be (A.M.); jaziri@ulb.ac.be (M.E.J.); marc.behr@ulb.ac.be (M.B.); 2 Laboratoire de Biotechnologie Végétale et Amélioration des Plantes (LABAP), Université Abdou Moumouni de Niamey, BP 10727 Niamey, Niger; 3 Department of Plant Biotechnology and Bioinformatics, Ghent University, 9052 Ghent, Belgium; gegoe@psb.vib-ugent.be (G.G.); woboe@psb.vib-ugent.be (W.B.); 4 VIB Metabolomics Core, 9052 Ghent, Belgium; 5 VIB Center for Plant Systems Biology, 9052 Ghent, Belgium; 6 Unit of Therapeutic Chemistry and Pharmacognosy, Université de Mons, 7000 Mons, Belgium; pierre.duez@umons.ac.be; 7 Unité de Glycobiologie Structurale et Fonctionnelle, Univ. Lille, CNRS, UMR 8576—UGSF, F-59000 Lille, France; simon.hawkins@univ-lille.fr (S.H.); godfrey.neutelings@univ-lille.fr (G.N.); 8 Biotechnology of Natural Products, Technische Universität München, 85354 Freising, Germany; thomas.hofmann@tum.de (T.H.); wilfried.schwab@tum.de (W.S.)

**Keywords:** UGT72 family, poplar, monolignol glucosides, lignin, vascular tissues, gene expression, glycosyltransferases

## Abstract

Monolignols are the building blocks for lignin polymerization in the apoplastic domain. Monolignol biosynthesis, transport, storage, glycosylation, and deglycosylation are the main biological processes partaking in their homeostasis. In *Arabidopsis thaliana*, members of the uridine diphosphate-dependent glucosyltransferases UGT72E and UGT72B subfamilies have been demonstrated to glycosylate monolignols. Here, the poplar UGT72 family, which is clustered into four groups, was characterized: Group 1 UGT72AZ1 and UGT72AZ2, homologs of Arabidopsis UGT72E1-3, as well as group 4 UGT72B37 and UGT72B39, homologs of Arabidopsis UGT72B1-3, glycosylate monolignols. In addition, promoter-GUS analyses indicated that poplar *UGT72* members are expressed within vascular tissues. At the subcellular level, poplar UGT72s belonging to group 1 and group 4 were found to be associated with the nucleus and the endoplasmic reticulum. However, UGT72A2, belonging to group 2, was localized in bodies associated with chloroplasts, as well as possibly in chloroplasts. These results show a partial conservation of substrate recognition between Arabidopsis and poplar homologs, as well as divergent functions between different groups of the UGT72 family, for which the substrates remain unknown.

## 1. Introduction

Glycosyltransferases (GT) (EC 2.4.x.y) are defined as enzymes that utilize an activated donor sugar substrate that contains a (substituted) phosphate leaving group [1]. Glycosylation can modify the biochemical properties and/or the subcellular localization of a wide range of substrates, such as proteins, lipids, hormones, or phenylpropanoid compounds [2,3]. Based on available genome sequences, GT are ubiquitous enzymes, and 110 families have been identified so far, from which 42 are present in both model plants *Arabidopsis thaliana* and *Populus trichocarpa* (http://www.cazy.org/GlycosylTransferases.html [4]). GT1, comprising the uridine diphosphate glycosyltransferases (UGT) that use uridine diphosphate (UDP)-activated sugars as donor molecules, is the largest GT family in plant [5]. The number of UGT has been estimated to be 122 (out of 497 GT) in *A. thaliana* and 281 (out of 788 GT) in *P. trichocarpa* [4]. UGT (E.C. 2.4.1.x) share a conserved motif, the Plant Secondary Product Glycosyltransferase (PSPG) box, consisting of 44 aa at the C-terminal part of the protein and which is involved in the binding of the UDP-sugar molecule [6]. UGTs glycosylate a large array of secondary metabolites, such as terpenoids, alkaloids, steroids, and flavonoids, as well as phenylpropanoids [7,8].

**Figure 1:**
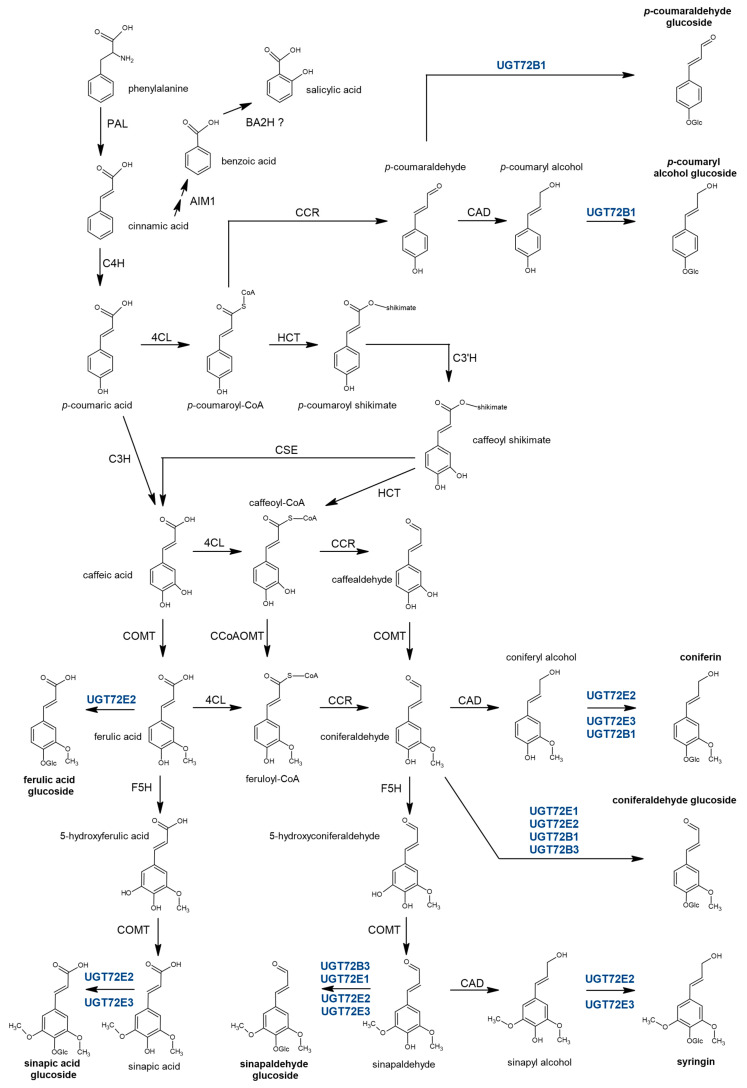
The monolignol biosynthetic pathway with associated glycosylated products and uridine diphosphate glucosyltransferases (UGT) catalyzing in vitro this glycosylation in Arabidopsis (indicated in blue), in presence of uridine diphosphate (UDP)-glucose (references within the text). UGT72E1 has been localized in the nucleus [32]. PAL-dependent salicylic acid biosynthetic pathway is also represented [33]. Glycosylated molecules are represented in bold. 4CL, 4-coumarate ligase; AIM1, abnormal inflorescence meristem 1; BA2H, benzoic acid 2-hydroxylase; C3H, 4-coumarate 3-hydroxylase; C3′H, 4-coumaroyl shikimate/quinate 3′-hydroxylase; C4H, cinnamate-4-hydroxylase; CAD, cinnamyl alcohol dehydrogenase; CCoAOMT, caffeoyl-CoA 3-*O*-methyltransferase; CCR, cinnamoyl CoA reductase; COMT, caffeate *O*-methyltransferase; CSE, caffeoyl shikimate esterase; F5H, ferulate 5-hydroxylase; HCT, hydroxycinnamoyl-CoA:shikimate/quinate hydroxycinnamoyltransferase; PAL, phenylalanine ammonia-lyase.

Monolignols (or (hydroxyl)cinnamyl alcohols), i.e., *p*-coumaryl alcohol, coniferyl alcohol, and sinapyl alcohol, are the main building blocks of lignin and (neo)lignans [9]. When glycosylated by UGT, in the presence of UDP-glucose, these phenylpropanoids give rise to *p*-coumaryl alcohol glucoside, coniferin, and syringin, respectively (Figure 1). Monolignol glucosides accumulate in vascular tissues, including phloem, cambial tissue, and differentiating xylem, of both conifers and angiosperms, and they can be incorporated into the lignin polymer, as shown by experiments following injection of radio-labeled glycosylated monolignols in stems [10,11,12,13,14,15,16,17,18,19,20].

Glycosylation of monolignols renders them less cytotoxic by reducing their reactivity [21]. Monolignol glucosides presumably serve as storage or transport forms of monolignols [22,23], and their occurrence in vacuoles has been demonstrated [24]. Monolignols have been shown to be transported through the plasma membrane and monolignol glucosides through the tonoplast in the presence of ATP, indicating that their transport is ensured by ATP-binding cassette-like transporters that carry them to the cell wall or the vacuole, respectively [25]. In accordance, a *p*-coumaryl alcohol plasma membrane transporter, AtABCG29, has been identified and characterized in Arabidopsis [26]. However, as predicted by Vermaas et al. [27] by molecular dynamics simulation, the transport of monolignols to the apoplastic space is likely to occur also by passive diffusion across the plasma membrane.

In Arabidopsis, several recombinant proteins belonging to the UGT72 family, including UGT72E1-3 [28,29], as well as UGT72B1 and UGT72B3 [30], have been found to be able to glucosylate in vitro cinnamic acids, cinnamaldehydes and cinnamyl alcohols (Figure 1) with different affinities. More precisely, UGT72E1-3 may 4-*O* glucosylate coniferaldehyde and sinapaldehyde, as well as coniferyl and sinapyl alcohols with different specificities. UGT72E2 and UGT72E3 also accept ferulic and sinapic acids as substrates [29]. UGT72B1 displayed an activity with *p*-coumaryl, coniferyl and dihydroconiferyl alcohols, coniferaldehyde, and *p*-coumaraldehyde, while UGT72B3 could glucosylate coniferaldehyde and sinapaldehyde [30]. In contrast, UGT72C1, UGT72D1, and UGT72B2 had no activity towards these substrates [28,30]. An accumulation of coniferin and syringin, as well as ferulic acid 4-*O*-glucoside and sinapic acid 4-*O*-glucoside, was measured in Arabidopsis overexpressing *UGT72E* genes, with specificities depending on the gene overexpressed [31]. In contrast to lines with up- or down-regulation of *UGT72E1-3*, for which no lignin-associated phenotype and/or lignin modification (qualitative and quantitative) was reported, the *ugt72b1* mutant was characterized by an ectopic lignification in floral stems, along with arrested growth [30]. When compared to the wild type (WT), both the *ugt72b1* mutant and transgenic Arabidopsis lines overexpressing *UGT72B1* had an increase in coniferin [30]. As suggested by these authors, the origin of the higher amount of coniferin in the *ugt72b1* mutant is possibly related to the increased expression of both *UGT72B3* and *UGT72E2* that was detected in this mutant.

Overall, these reports indicate that different recombinant glycosyltransferases from the Arabidopsis UGT72 family have different substrate specificities in vitro, suggesting that they are all involved in a complex regulatory network required for adequate maintenance of the monolignol/monolignol glucoside pool, as well as that of their acid or aldehyde precursors (Figure 1).

The aim of the present study was to characterize the UGT72 family in poplar (*P. tremula* × *P. alba* clone INRA 717-1B4), a woody perennial plant, to identify which members are involved in the glycosylation of monolignols. Our results showed that poplar UGT72 family members clustered into four groups. Enzymatic activities of each member, tested with 11 different phenylpropanoid substrates, as well as analysis of glycosylated monolignols in transgenic poplars overexpressing the corresponding genes, allowed the identification of 4 poplar UGT72s glycosylating monolignols, including members of group 1 (UGT72AZ1 and UGT72AZ2) and group 4 (UGT72B37 and UGT72B39). The poplar UGT72s belonging to group 2 and 3 had no activity towards the tested substrates. Analysis of promoter-GUS expression profiles showed expression in vascular tissues for all genes analyzed. Finally, differences in subcellular localization were found for the poplar UGT72s. Altogether, these data suggest different functions for members of the UGT72 family in poplar.

## 2. Results

### 2.1. Cloning of the Full Length cDNA of Poplar *UGT72* Genes

Ten sequences homologous to Arabidopsis *UGT72*s were identified in the *P. trichocarpa* genome sequence. Primers to obtain *P. tremula* × *P. alba* orthologous sequences were designed for 8 of these genes (Table S1). Potri.007G029800 was not cloned because its coding sequence was 99% identical to that of Potri.007G030300 (*UGT72AZ1*), and Potri.014G041900 was not cloned as it has a truncated PSPG motif, and the catalytically active His at the N-terminus is missing (Figure S1). None of these genes had introns. The *P. tremula* × *P. alba UGT72* genes were named *UGT72A2*, *UGT72AZ1*, *UGT72AZ2*, *UGT72B36*, *UGT72B37*, *UGT72B38*, *UGT72B39*, and *UGT72BB1*, according to the UGT Nomenclature Committee (https://prime.vetmed.wsu.edu/resources/udp-glucuronsyltransferase-homepage). An alignment of the different deduced polypeptide sequences of UGT72 members of *P. tremula* × *P. alba* and Arabidopsis shows the PSPG domain and conserved key residues in these sequences (Figure S1). The percentages of identity between poplar UGT72s and their Arabidopsis homologs is given in Table S2, and their respective polypeptide length, predicted pI, and predicted molecular weight in Table S3.

**Figure 2:**
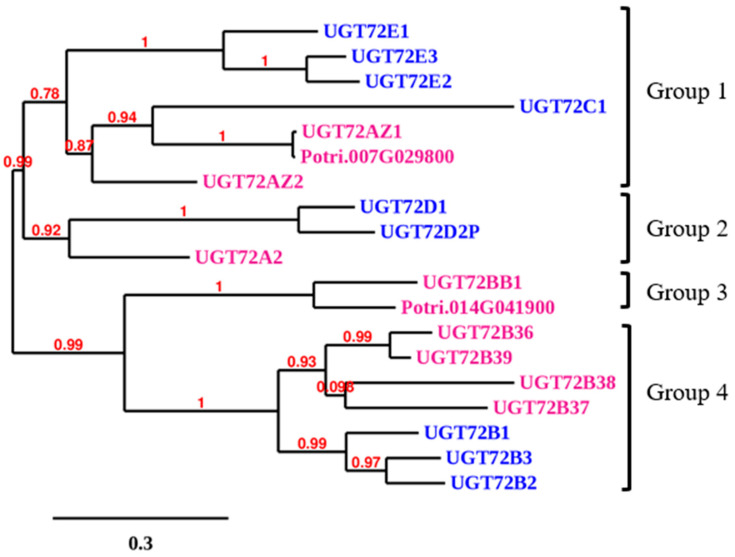
Phylogenetic tree of UGT72s from Arabidopsis and poplar. This tree was generated by the approximate likelihood-ratio test method. Arabidopsis and poplar polypeptides are shown in blue (as in Figure 1) and magenta, respectively. Scale bar: expected numbers of amino acid substitutions per site. Protein alignment is given in Figure S1.

As shown in Figure 2, the analysis of the phylogenetic relationships between UGT72s of Arabidopsis and poplar revealed that the proteins clustered into 4 different groups. The first group comprises the Arabidopsis UGT72E1–E3 and UGT72C1, as well as the poplar UGT72AZ1–AZ2. The second group is made up of the Arabidopsis UGT72D1–D2P and the poplar UGT72A2. The third group contains UGT72BB1 with no Arabidopsis homolog, and the fourth group includes UGT72B members from both Arabidopsis (B1–B3) and poplar (B36–B39).

### 2.2. Recombinant UGT72B37 and UGT72B39 Proteins Glycosylate Monolignols

**Figure 3:**
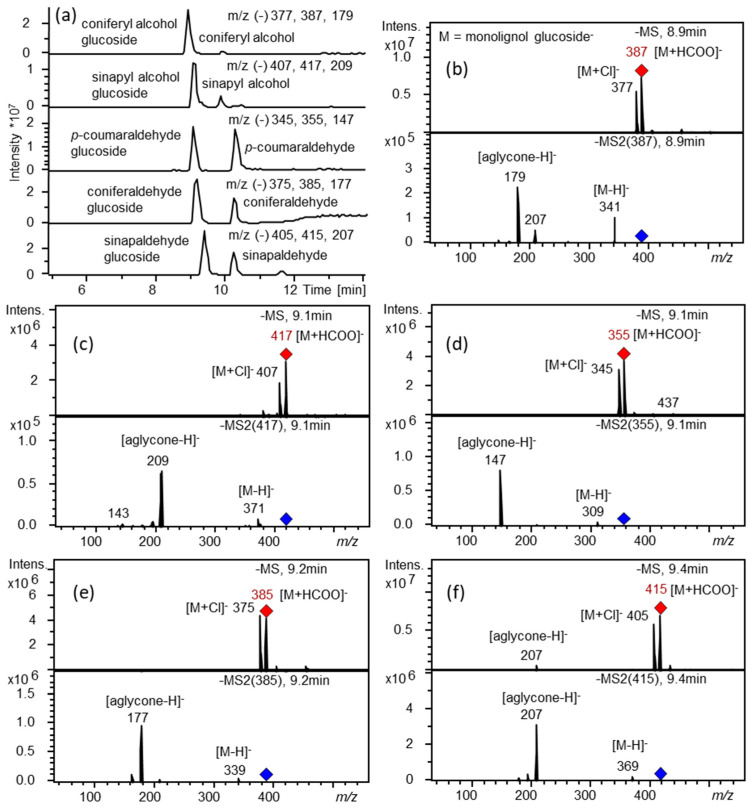
LC-MS analysis of UGT72B37 enzymatic reaction products. Extracted ion chromatograms (**a**), mass spectra (MS in red) and product ion spectra (MS2 in blue) of compounds formed by UGT72B37 with coniferyl alcohol (**b**), sinapyl alcohol (**c**), *p*-coumaraldehyde (**d**), coniferaldehyde (**e**), and sinapaldehyde (**f**) as substrates. UDP-glucose was used as sugar donor. M, monolignol glucoside.

The 8 poplar *UGT72s* were cloned in the pGEX-4T-1 expression vector containing an N-terminal glutathione S-transferase (GST-fusion) tag. After production in *E*. *coli* and purification, recombinant proteins were tested using 11 substrates of the phenylpropanoid/monolignol pathway (Figure 1) using UDP-glucose as sugar donor. As shown in Table 1, as well as in Figure 3 and Figure S2, recombinant UGT72AZ2, UGT72B37, and UGT72B39 recognized at least one substrate in vitro, whereas no activity towards any of the tested substrates was detected for the 5 other UGT72s. UGT72B37 (group 4) showed a broad substrate specificity as it could glycosylate *p*-coumaraldehyde, coniferaldehyde, sinapaldehyde, coniferyl alcohol, and sinapyl alcohol (Figure 3), while UGT72B39 (group 4) only glycosylated coniferyl alcohol (Figure S2), and UGT72AZ2 (group 1) glycosylated ferulic and sinapic acids (Figure S2).

### 2.3. Accumulation of Monolignol Glycosides in Transgenic Poplar Overexpressing *UGT72s*

Transgenic overexpression poplar lines were produced for all members of the *UGT72* family, except for *UGT72B38*, for which no plants could be regenerated. Overexpression of each *UGT72* in these lines was confirmed by RT-qPCR on leaves of plants cultivated for 4 months in soil. As shown in Figure S3, the level of overexpression was variable for the different constructs, being about 1000–8000-fold for 35S-UGT72AZ1 and 35S-UGT72AZ2 lines (group 1), 2500- to 4500-fold for the 35S-UGT72BB1 lines (group 3), and 200–900-fold in 35S-UGT72A2 lines (group 2), whereas it reached 10- to maximum 70-fold in the 35S-UGT72B36, 35S-UGT72B37, and 35S-UGT72B39 (group 4) lines. The differences between the overexpression levels among the different *UGT72* genes might be related to the endogenous level of expression of these genes in leaves, which seems inversely related with the fold-increased level. Indeed, in silico analysis of gene expression in different tissues of 1-year-old *P. trichocarpa* based on the data published by Shi and colleagues [34] shows that *UGT72AZ1-2* (group 1) and *UGT72BB1* (group 3) are expressed at low levels in leaves (transcript abundance between 2 and 8), whereas *UGT72A2* (group 2) shows an intermediate expression (transcript abundance of approximately 150) compared to *UGT72B36-39* (group 4), which are highly expressed (transcript abundance between 150 and 1000) (Figure S4A).

**Figure 4:**
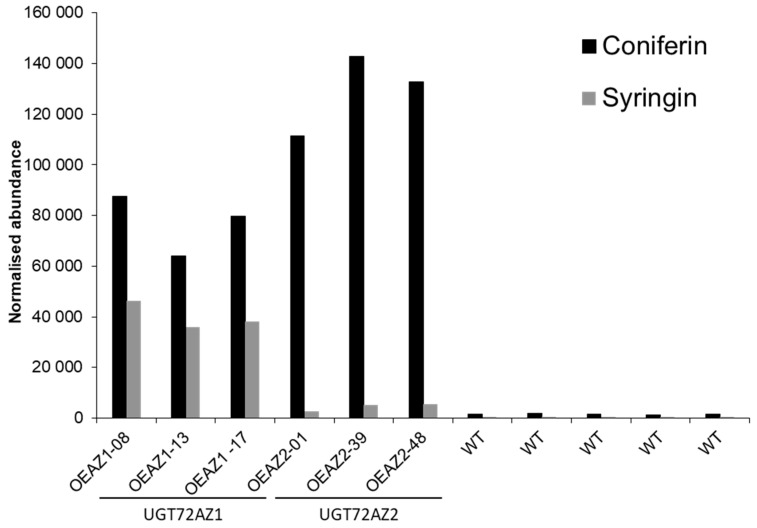
LC-MS analysis of coniferin and syringin in leaf of 4-month-old wild type (WT), *UGT72AZ1*, and *UGT72AZ2* overexpressing lines grown in the greenhouse. Measures were made in 5 independent WT and in 3 independent transgenic lines for each construct. This analysis was repeated with HPLC-UV with similar results.

Examination of the transgenic plants revealed that no phenotypical (at the whole plant level), nor anatomical differences (in transversal stem cross sections stained with phloroglucinol) could be observed in any of the different poplar *UGT72* overexpressing lines. To further characterize these lines, we investigated the profiles of glycosylated phenolics in leaf extracts in order to identify the in vivo acceptor substrates. LC-MS analysis (Figure 4) indicated that the lines overexpressing the group 1 genes accumulated coniferin and syringin (*UGT72AZ1*), or just coniferin (*UGT72AZ2*). Traces of coniferin were detected in the WT, while syringin was below the detection level. Transgenic lines overexpressing group 2 or group 4 *UGT72* genes did not accumulate detectable amounts of both glycosides using a HPLC-UV system (no plant material was available for group 3).

To assess whether the observed increase in the quantity of coniferin and syringin levels in leaves was accompanied by modifications in lignin levels, we analyzed stem wood, stem bark, and root samples from 35S-UGT72AZ1 and 35S-UGT72AZ2 lines. Our results indicated that lignin content, as measured by the acetyl bromide method, was not affected in any of the samples from these lines (Figure S5).

### 2.4. *UGT72s* Expression Profiles in Poplar

**Figure 5:**
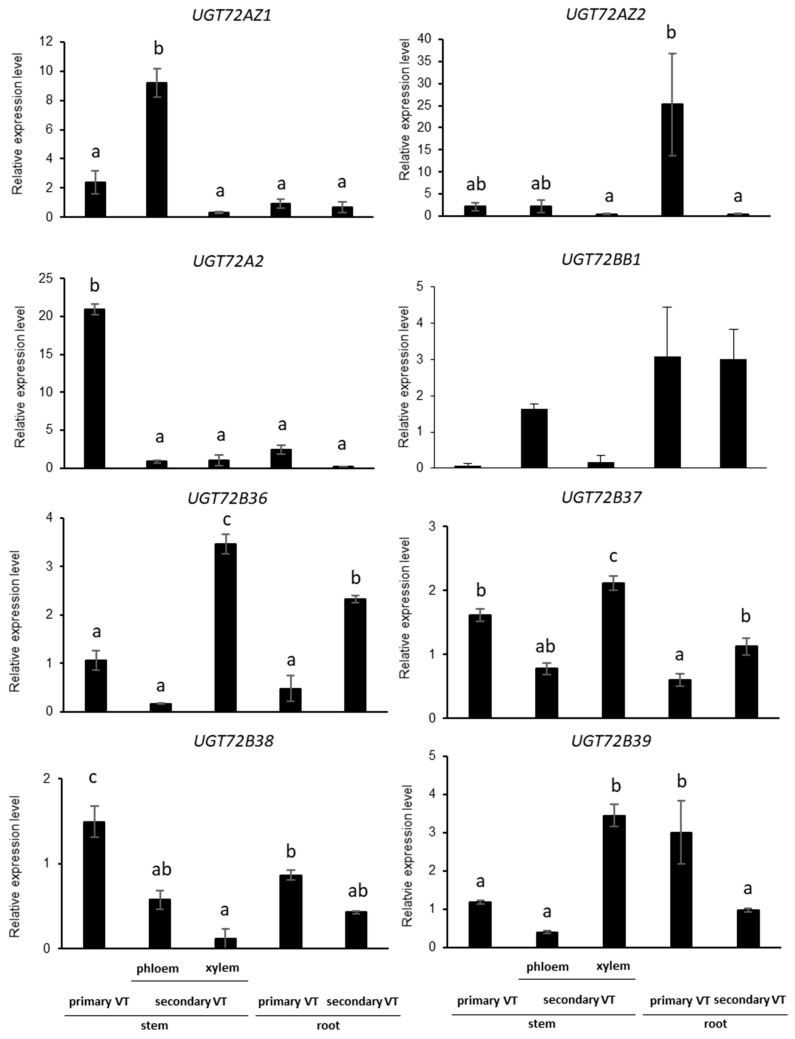
RT-qPCR expression profile of *UGT72s* in different parts of 4-month-old poplar trees grown in the greenhouse. Normalized relative expressions are means of 3 biological replicates (± SE). Significantly different values (*p* < 0.05) are represented by different letters (ANOVA and Tukey post hoc test). VT, vascular tissue.

To further characterize the different poplar *UGT72* genes, their expression profile was analyzed by RT-qPCR in different parts of the stem and the root of 4-month-old greenhouse grown WT poplar. Primary vascular tissues (primary VT) correspond to the first 2 cm below the apex. Secondary vascular tissues (secondary VT) were sampled 50 cm below the apex and separated into bark and wood (entitled phloem and xylem in Figure 5, respectively). Young root with primary VT and old root with secondary VT (~1 cm diameter) were also investigated. As shown in Figure 5, expression of the eight genes was detected in all samples but at different levels. *UGT72AZ1* was principally expressed in the phloem of the stem, *UGT72AZ2* in young roots, *UGT72A2* in young stems, *UGT72BB1* in the phloem of the stem and in roots (thus possibly in the phloem of the root), and *UGT72B36* in xylem of the stem and in old roots (thus possibly in the xylem of root), while *UGT72B37* was expressed in all parts with the highest level in stem secondary xylem, *UGT72B38* in young stems and roots (thus possibly in primary VT), and *UGT72B39* in the xylem of the stem and in young roots (probably in the xylem of the root).

### 2.5. *UGT72s* Are Expressed within Vascular Tissues

**Figure 6:**
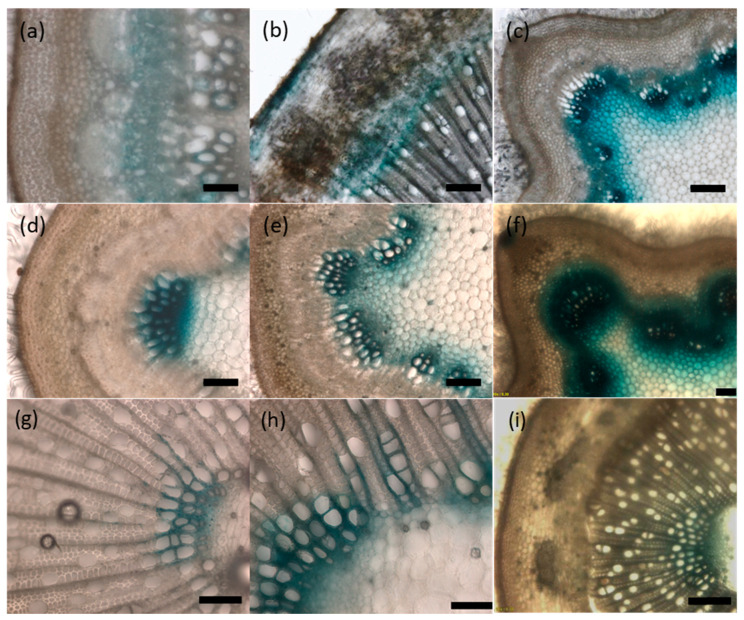
*GUS* expression patterns in tissues of 4-month-old poplar grown in the greenhouse. (**a**) *pUGT72AZ1::GUS* expression in the stem 10 cm below the apex. (b) *pUGT72AZ2::GUS* expression in 4 mm diameter root. (**c**) *pUGT72A2::GUS* expression in stem 5 cm below the apex. (**d**) *pUGT72B36::GUS* expression in stem 10 cm below the apex. (**e**) *pUGT72B37*::*GUS* expression in stem 10 cm below the apex. (**f**) *pUGT72B39*::*GUS* expression in stem 5 cm below the apex. (**g**) *pUGT72B36*::*GUS* expression in stem 30 cm below the apex. (**h**) *pUGT72B37*::*GUS* expression in stem 30 cm below the apex. (**i**) *pUGT72B39*::*GUS* expression in stem 30 cm below the apex. Scale = 100 µm (**a**,**c**,**f**,**i**), 200 µm (**b**,**d**,**e**,**g**,**h**).

In order to confirm the expression data obtained by RT-qPCR and provide more detailed information about *UGT72s* spatial expression, histochemical GUS staining of *pUGT72::GUS* transgenic poplar lines was investigated. Unfortunately, no plants could be regenerated for the *pUGT72B38*::*GUS* and *pUGT72BB1::GUS* constructs. A focus was made on the organs where the expression measured by RT-qPCR of the respective genes was most pronounced (i.e., root for *UGT72AZ2* and stem for *UGT72A2*, *UGT72B36*, *UGT72B37*, and *UGT72B39*). As shown in Figure 6 and Figure S6, the 6 *UGT72* genes are expressed in vascular tissues with different specificities. *UGT72AZ1* is expressed mainly in the phloem within the stem and leaf (Figure 6a and Figure S6b), *UGT72AZ2* in the cortical region, as well as in the phloem and differentiating xylem of the root (Figure 6b), *UGT72A2* (Figure 6c), and *UGT72B39* (Figure 6f,i) in the primary xylem of stem and *UGT72B36* (Figure 6d,g) and *UGT72B37* (Figure 6e,h) within the stem xylem. These results are in accordance with the RT-qPCR results (Figure 5) and with RNA-Seq data obtained from different cross sections within vascular tissues of the stem of a 45-year-old *P. tremula* (Figure S4b, heatmap made with data from group 4 taken from the AspWood database [35]).

**Figure 7:**
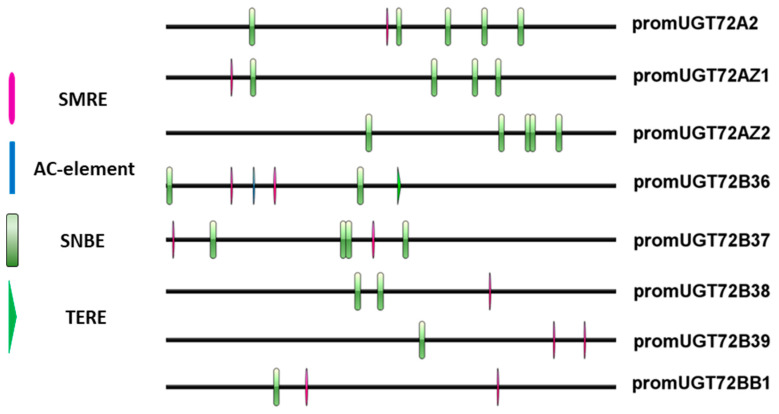
Secondary cell wall-related cis-elements in the promoters of poplar *UGT72* members. SMRE, Secondary wall MYB-Responsive Element. SNBE, Secondary wall NAC-Binding Element. AC-element, R2R3MYB Responsive Element. TERE, Tracheary-Element-Regulating *cis*-Element. The *cis*-element sequences and the associated binding factors in Arabidopsis are listed in Table S4. The analysis was made on the 1500 bp upstream the coding sequences.

In silico *UGT72s* promoter analysis (Figure 7 and Tables S4 and S5) reveals the occurrence of *cis*-elements involved in the differentiation of vascular tissues, such as Secondary wall MYB-Responsive Element (SMRE), Secondary wall NAC-Binding Element (SNBE), R2R3MYB Responsive Element (AC-elements), and Tracheary-Element-Regulating *cis*-Element (TERE) [36,37]. SNBE were present in all promoters (from one element in *pUGT72B39-BB1* to five elements in *pUGT72A2*/*AZ2*). TERE is found in several genes related to xylem differentiation [38] and was detected only in *pUGT72B36*. Several SMRE and AC-elements were detected in *pUGT72BB1*, *pUGT72B36*, *pUGT72B37*, and *pUGT72B39*, while *pUGT72B38*, *pUGT72AZ1*, and *pUGT72A2* displayed a single SMRE, and *pUGT72AZ2* did not exhibit any of these *cis*-elements. The presence of these *cis*-elements may explain the preferential expression of the poplar *UGT72* genes in vascular tissues (Figure 5 and Figure 6).

### 2.6. Proteins of Members of The Poplar UGT72 Family Preferentially Accumulate in Vascular Tissues

**Figure 8:**
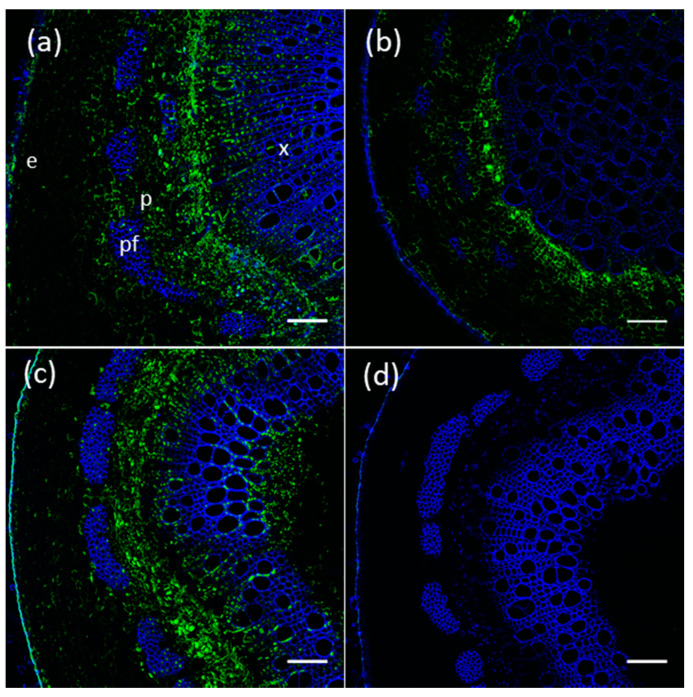
Immunolocalization of UGT72AZ1, UGT72AZ2, and UGT72A2 in transversal sections of 3-month-old WT poplar grown in the greenhouse. (**a**) UGT72AZ1 in the stem at 20 cm below the apex. (**b**) UGT72AZ2 in the root. (**c**) UGT72A2 in the stem at 10 cm below the apex. (**d**) Negative control in stem (no primary antibody). Alexa 488 signal corresponding to the UGT72 epitope is shown in green. Lignin autofluorescence is in blue. e, epidermis; p, phloem; pf, phloem fiber; x, xylem. Scale = 100 µm.

The immunolocalization of UGT72AZ1, UGT72AZ2 (group 1) and UGT72A2 (group 2), for which custom antibodies were designed, was investigated in *P. tremula* × *P. alba* grown in the greenhouse (for antibody characterization, see Figure S7). Based on GUS histochemical staining and RT-qPCR results, immunolocalizations were performed in stem for UGT72AZ1 and UGT72A2 and in root for UG72AZ2. As shown in Figure 8, UGT72AZ1 was localized mainly in the phloem (Figure 8a), the cambium and the developing xylem of the stem, while UGT72AZ2 was located mainly in the cambium, as well as in the cortex of the root (Figure 8b). UGT72A2 was principally detected in the phloem but also in the differentiating xylem and in ray cells of the xylem, as well as in the pith (Figure 8c). These results indicate that UGT72AZ1, UGT72AZ2, and UGT72A2 seem to preferentially accumulate in vascular tissues, albeit with different patterns.

### 2.7. Subcellular Localization of the Poplar UGT72s

**Figure 9:**
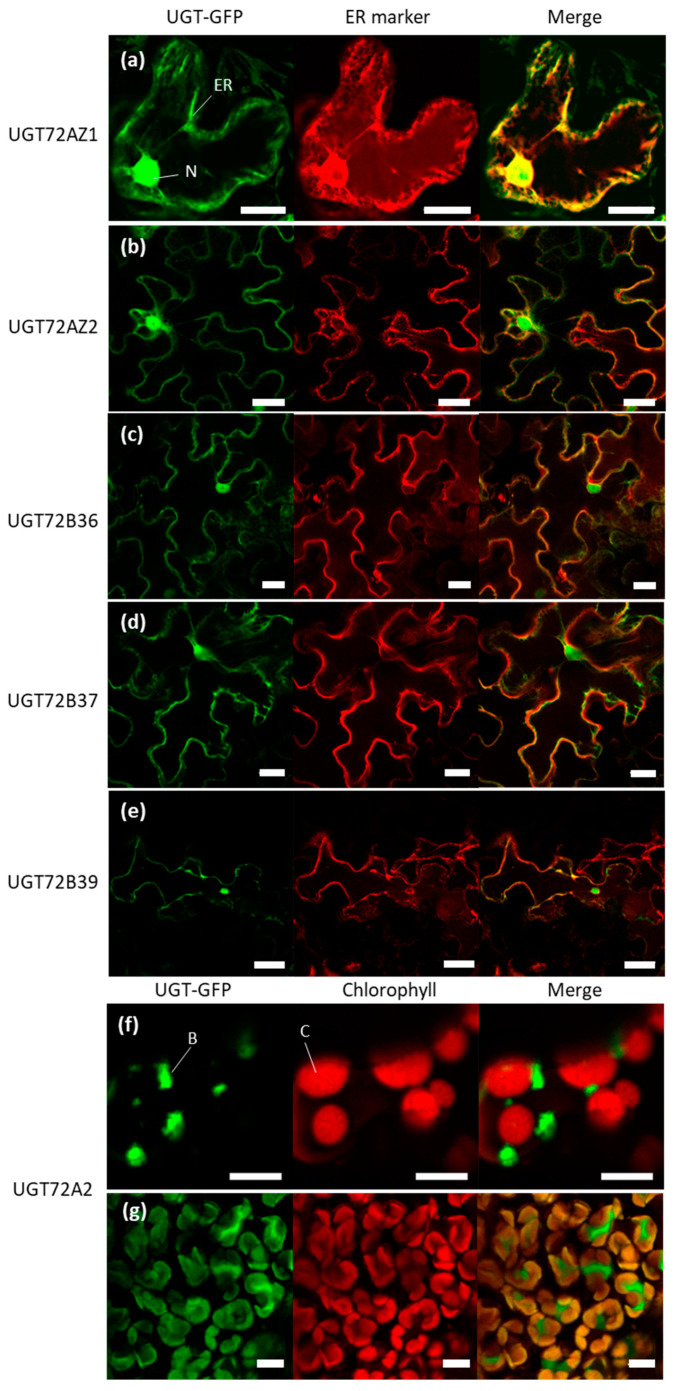
Subcellular localization of poplar UGT72 fused to GFP. (**a**–**f**) UGT72-GFP fusion proteins and 35S::AtWAK2-mCherry, as ER marker (**a**–**e**) in *N*. *benthamiana* leaf epidermal cells after agroinfiltration. UGT72A2-GFP (**f**) was localized in bodies associated to chloroplasts (red fluorescence). (**g**) Stable transgenic poplar line expressing the same *35S::UGT72A2-GFP* construct. UGT72A2 was localized in in bodies associated with chloroplasts and possibly in chloroplasts. Images are representative of biological triplicates. B, bodies associated with chloroplast; C, chloroplast; ER, endoplasmic reticulum; N, nucleus. Scale = 20 µm (**a**), 35 µm (**b**–**d**), 70 µm (**e**), 5 µm (**f**), 10 µm (**g**).

To investigate the subcellular localization of poplar UGT72 members, C-terminal GFP-fused UGTs were transiently expressed in tobacco leaf epidermis using agroinfiltration, together with an endoplasmic reticulum (ER) marker. As shown in Figure 9, five UGT72s (UGT72AZ1 and UGT72AZ2 from group 1 and UGT72B36, UGT72B37, and UGT72B39 from group 4) showed a defined ER and nuclear localization (Figure 9a–e). In contrast, UGT72A2 (group 2) was localized in bodies associated with the chloroplasts (Figure 9f). The UGT72A2 localization was further investigated in stable transgenic poplars harboring the same *35S::UGT72A2-GFP* construct. In this case, the localization of UGT72A2-GFP was also observed in bodies associated with the chloroplasts and possibly within chloroplasts (Figure 9g).

## 3. Discussion

The aim of the present study was to characterize the *UGT72* family in poplar, a woody perennial plant, and to identify genes that could be involved in the glycosylation of monolignols. UGTs are known to show in vitro activities towards a large range of different substrates, and their substrate specificity cannot be predicted merely based on phylogenetic relationships [39]. As a first step in the characterization of the poplar *UGT72* family, a phylogenetic tree of Arabidopsis and poplar UGT72 members was generated allowing the identification of 4 groups in poplar, of which 3 were common with Arabidopsis (Figure 2).

### 3.1. Some Members of the Poplar UGT72 Family Are Involved in the Glycosylation of Monolignols

Group 1 contains the Arabidopsis UGT72E1-E3 and UGT72C1 proteins, as well as the poplar UGT72AZ1 and UGT72AZ2 proteins. Recombinant Arabidopsis UGT72E1 uses coniferaldehyde and sinapaldehyde; UGT72E2 is able to glycosylate coniferyl and sinapyl alcohols, as well as coniferaldehyde, sinapaldehyde, and ferulic and sinapic acids; UGT72E3 displays activity towards coniferyl and sinapyl alcohols and sinapic acid; and UGT72C1 was found to glycosylate caffeic acid [29,40,41]. In brief, UGT72E2 is able to glycosylate monolignols and monolignol precursors, and UGT72E1 and UGT72E3 seem to be redundant to UGT72E2, at least partially. Here, in poplar, no activity towards any of the tested substrates was detected for UGT72AZ1. In contrast, UGT72AZ2 was found to glycosylate ferulic and sinapic acids but not (hydroxy)cinnamaldehydes nor (hydroxy)cinnamyl alcohols. In agreement with these results, it was previously observed that the recombinant *Populus tomentosa* PtUGT1, which is orthologous to UGT72AZ2, also showed no activity towards monolignols. Although belonging to the same phylogenetic group, these data indicate that the substrate specificity of poplar group 1 UGT72s is different from their Arabidopsis counterparts.

Metabolic analyses by HPLC were previously made on Arabidopsis overexpressing *UGT72E1-E3* to measure the accumulation of glycosides of intermediates of the phenylpropanoid pathway. In the WT, only a low amount of coniferin was detected in rosette leaves [31]. In contrast, overexpression of *UGT72E1*, *UGT72E2*, and *UGT72E3* led to a 6-fold, 85-fold, and 56-fold increase in coniferin content, respectively. An accumulation of syringin, ferulic acid 4-*O*-glucoside, and sinapic acid 4-*O*-glucoside was also detected in the transgenic plants overexpressing *UGT72E2* and *UGT72E3* [31,42]. Similarly, our results showed that coniferin and syringin accumulated in 35S-UGT72AZ1 lines and coniferin in 35S-UGT72AZ2 lines. The differences observed between UGT72 members in substrate specificity in vitro and in vivo may be explained by the availability of the appropriate substrates *in planta* [2,43].

Group 2 includes the Arabidopsis UGT72D1 and UGT72D2P and the poplar UGT72A2. No activity towards cinnamic acids, cinnamaldehydes, nor cinnamyl alcohols has been found for UGT72D1 [28], and there is no data available for substrate specificity of UGT72D2P. The recombinant UGT72A2 protein did not use any of the tested phenylpropanoid substrates, and we did not observe increased accumulation of coniferin and syringin in the lines overexpressing *UGT72A2*. Further studies related to the identification of the substrates of these respective UGTs are needed to help to elucidate their function.

Group 3 comprises the poplar UGT72BB1, for which there is no corresponding Arabidopsis member. UGT72BB1 showed no activity towards the phenylpropanoids tested.

Finally, group 4 includes Arabidopsis UGT72B1-B3 and the poplar UGT72B36-B39. The recombinant UGT72B1 was reported to have glycosylating activity for *p*-coumaryl alcohol, coniferyl alcohol, dihydroconiferyl alcohol, *p*-coumaryl aldehyde, and coniferaldehyde. No activity was demonstrated for UGT72B2 towards the phenylpropanoids tested, and UGT72B3 showed activity on coniferaldehyde and sinapaldehyde [30]. Amongst the different members of the poplar UGT72 family, recombinant UGT72B37 glycosylated the highest number of substrates, including coniferyl and sinapyl alcohols, *p*-coumaraldehyde, coniferaldehyde, and sinapaldehyde, whereas UGT72B39 only used coniferyl alcohol. Based on the substrate specificities, UGT72B37 shows an enzymatic activity that covers that of Arabidopsis UGT72B1 and UGT72B3. However, in contrast to transgenic Arabidopsis lines overexpressing *UGT72B1* that accumulate a higher amount of coniferin (up to 2-fold when compared to the WT [30]), no increase in the levels of monolignol glycosides were detected in leaves of transgenic poplar overexpressing *UGT72B37* and *UGT72B39*.

### 3.2. UGT72s Are Expressed in the Vascular System

The *GUS* expression driven by the poplar *UGT72* promoters was observed in the vascular tissues for the investigated genes (Figure 6 and Figure S6). In Arabidopsis, *UGT72* members are also expressed within vascular tissues. Concerning group 1, *pUGT72E1* was found to be active in the root and *pUGT72E2*, as well as *pUGT72E3*, were active in whole seedlings, in leaves, and in floral organs [31]. No data are available for *pUGT72C1*. In poplar, *pUGT72AZ1* was active mainly in the phloem of the stem and *pUGT72AZ2* in the cambial zone of the root (Figure 6), and their corresponding proteins had a similar localization, as analyzed by immunolocalization (Figure 8).

For group 2, Arabidopsis *pUGT72D1* was found to drive expression in phloem and in xylem [44]. In this study, poplar *pUGT72A2* was also active in phloem and xylem, and the corresponding protein accumulated correspondingly.

For group 4, in Arabidopsis, GUS staining of *pUGT72B1*::*GUS* plants was found mainly in the xylem of the upper part of the stem and only in the xylem for older stem. For *pUGT72B2*, GUS staining was mainly detected in veins of cotyledons and leaves and, for *pUGT72B3* in basal petiole, young leaf margins and slightly in the floral stem [30]. In poplar, the expression driven by the *pUGT72B36*, *pUGT72B37*, and *pUGT72B39* was found mainly in the primary xylem within the stem (Figure 6).

In silico analysis of the promoters revealed the presence of key *cis*-elements involved in vascular differentiation, such as SMRE, SNBE, AC-elements, and TERE [37,45,46,47]. The promoters of the *UGT72B36-39* genes that were highly expressed in the xylem (Figure 5 and Figure S4) have several *cis*-elements related to xylem development, suggesting a possible role of these elements in their tissue-specific expression. In poplar, PtrMYB2, PtrMYB3, PtrMYB20, and PtrMYB21 are orthologous to Arabidopsis MYB46 and MYB83 [48,49]. They bind to the consensus SMRE domain ACC(A/T)A(A/C)(T/C), which is found in the promoter of genes involved in cellulose, xylan, and lignin biosynthesis, as well as in *pUGT72BB1*, *pUGT72B36*, *pUGT72B37*, and *pUGT72B39* (Figure 7). In Arabidopsis, MYB46 and MYB83 transcription factors also induce the expression of downstream transcription factors (*MYB52*, *MYB63*) and genes involved in secondary cell wall formation [50]. Some of these SMRE are identical to AC-elements, which are known to induce the expression of lignin biosynthesis genes [50]. The fact that the poplar *UGT72B* promoters contain elements recognized by these transcription factors, therefore, suggests that they are part of the transcriptional programme leading to secondary cell wall formation in xylem. Besides, single or several SNBE were also found in *pUGT72s*. These elements are targeted by NAC transcription factors, in which downstream-regulated genes partially overlap with MYB-induced genes involved in secondary cell wall formation [46,51], such as *CesA4* and *CesA8* [38,52]. NAC master transcription factors regulating secondary cell wall formation in Arabidopsis vessels and fibers include VND6, VND7, SND1, NST1, and NST2, which bind to the SNBE to enhance the expression of downstream transcription factors and biosynthesis genes [53]. SNBE are also found in genes expressed during xylem programmed cell death, such as Arabidopsis *XYLEM CYSTEINE PEPTIDASE 1* and *2* [38] and poplar *METACASPASE 13* and *14* [54]. Such genes are mainly induced by VND6 and VND7 [38,46]. Although the VND6/VND7 activation of genes carrying a TERE in the promoter region remains debated [38,46,53,54,55], this *cis*-element is enriched in genes involved in tracheary element differentiation, comprising cell death [38]. Our data therefore suggest that poplar *UGT72s* may also play a role during the late phase of xylem differentiation. We performed a similar analysis on promoters of Arabidopsis *UGT72s* (Table S5). Overall, they display the same *cis*-elements, suggesting that they may be regulated by the same set of MYB and NAC transcription factors. *pUGT72D1* and *pUGT72E1* each displayed six SNBE, while only one to three were detected in other Arabidopsis *pUGT72s*. Six *cis*-elements regulated by secondary cell wall-MYBs were present in *pUGT72B3*, possibly suggesting a different regulation.

### 3.3. No Direct Impact of Poplar UGT72s Overexpression on Lignification Was Demonstrated

Previously, two studies have shown that modifications in the expression level of particular members of the *UGT72* family impact lignification. First, the ectopic expression of the *P. tomentosa PtUGT1* in tobacco induced an increase in approximately 60% in the Klason lignin amount in the stem [56]. As no enzymatic activity towards monolignols was found for the recombinant PtGT1, the effect of the overexpression of the corresponding gene on lignification was suggested to be probably indirect [56]. Second, the *ugt72b1* Arabidopsis mutant exhibits ectopic lignification [30]. The RNA-Seq analysis of the *ugt72b1* mutant showed modification of the expression of genes involved in the phenylpropanoid, flavonoid, and anthocyanin metabolisms. Particularly, genes involved in monolignol metabolism, possible monolignol transport (ABC transporters), lignin polymerization, as well as regulation of secondary cell wall formation, were upregulated in this mutant, indicating feedback regulation upon disturbance of monolignol glucosylation on whole lignin synthesis, by a mechanism that awaits further discovery [30]. In the light of the large transcriptome reprogramming in the *ugt72b1* mutant, the ectopic lignification phenotype is probably an indirect consequence of the mutation. This transcriptional shift is likely gene- and species-dependent, which may explain the absence of phenotype in lines overexpressing poplar *UGT72* members (Figure S5). Further evidence for indirect effect of mutation on lignification is that both *UGT72B1* overexpressor and *ugt72b1* mutant lines produce increased amount of coniferin [30]. A role for *UGT72s* in defense response may be hypothesized. Actually, 35S-UGT72E2 Arabidopsis plants were more resistant to the fungus *Verticillum longisporum* than the WT, which was attributed to the accumulation of coniferin [57]. Based on an RNA-Seq analysis, *UGT72E1* and *UGT72E2*, together with *UGT71C1*, were found to be the most upregulated *UGT* genes in Arabidopsis in response to infection by the fungus *Plasmodiophora brassicae* [58], suggesting that the corresponding proteins might be directly or indirectly involved in pathogen resistance. Further analysis of responses to pathogen infection of transgenic poplar overexpressing *UGT72AZ1/AZ2* and *UGT72B37/B39* may help to unravel the role of glycosylated monolignols in disease resistance or in developmental lignification.

### 3.4. Differences in Subcellular Localization of Poplar UGT72s May Indicate Divergent Functions among the Different Members of the Same Family

At the subcellular level, poplar UGT72s from group 1 (UGT72AZ1-2) and group 4 (UGT72B36-37 and 39) were localized in the nucleus and associated with the ER (Figure 9). Accordingly, several nuclear localization signals (NLSs) were detected in all UGT72s from poplar (cNLS mapper [59]). This analysis indicated a double localization, both in the nucleus and in the cytoplasm (Table S6). Several enzymes of the monolignol biosynthesis pathway (Figure 1), such as C4H, C3H, F5H, and HCT, are, at least partly, associated with the ER [60,61]. UGT84A23-GFP, involved in gallic acid glycosylation, is also possibly targeted to the ER [62]. Monolignol homeostasis may therefore rely on the activity of UGTs which colocalize with monolignol enzymes to directly glycosylate some of the intermediate products of this metabolic pathway to circumvent potential toxicity. Such a mechanism has been suggested for a vanillin-specific UGT in order to maintain a content at sub-toxic levels in the cell [63]. Assembly and disassembly of a given metabolon, for instance, an UGT coupled to a P450 protein, would provide additional flexibility in specialized metabolite biosynthesis. As an illustration, soluble UGT85B1 was found to be recruited to the ER, in close vicinity to enzymes of the same biosynthetic pathway, to favor dhurrin biosynthesis in response to insect attack [64]. Besides, UGT72E1 was previously identified by yeast two-hybrid as an interacting partner of SIS8, a putative mitogen-activated protein kinase kinase kinase, and these proteins were both localize in the nucleus when transiently expressed in tobacco leaf cells [32].

In contrast, UGT72A2 (group 2) was found to be associated with chloroplastic bodies, as well as possibly localized within chloroplasts, suggesting a different function than the other UGT72s from poplar. More precisely, UGT72A2-GFP was localized in bodies associated with the chloroplast following tobacco transient transformation with *Agrobacterium*, while, in stable transgenic lines carrying the same construct, the signal was also detected inside chloroplasts. These bodies are usually observed when large quantities of proteins are present, either due to high expression driven by the *CaMV35S* promoter or when high quantities of plasmids are used for the transfection [65,66]. We may speculate that, in stable transgenic poplars, the biosynthesis of proteins is more regulated; therefore, UGT72A2 is restricted to its native compartment, i.e., chloroplast. Finally, TargetP analysis [67] in poplar UGT72s for the presence of N-terminal presequences targeting the protein to a specific compartment did not reveal any preferential localization.

## 4. Materials and Methods

### 4.1. Plant Material and Growth Conditions

Both WT and transgenic poplars (*Populus tremula* × *P. alba, clone INRA 717-1B4*) were grown aseptically or in the greenhouse, as described previously [68].

### 4.2. Cloning of UGT72s Coding and Promoter Sequences from P. Tremula × P. Alba

Arabidopsis UGT72s protein sequences were used to screen the *P. trichocarpa* genome sequence (https://phytozome.jgi.doe.gov/pz/portal.html). Ten polypeptides sharing ~ 45% identity with Arabidopsis UGT were identified. *P. trichocarpa* coding sequences were then used to identify corresponding *P. tremula* × *P. alba* coding sequences using BLASTN in the *P. tremula* × *P. alba* clone INRA 717-1B4 genome sequence (https://urgi.versailles.inra.fr/Species/Forest-trees/Populus/Clone-INRA-717-1B4). *P. tremula* × *P. alba* gene specific primers were designed using PerlPrimer (v1.1.21) (Table S1) to amplify either corresponding cDNA or approximately 2 kb (1.6–2.1 kb depending on the gene) DNA region upstream of the start codon. The different *UGT72*s’ coding and promoter sequences were then cloned in pCR4 TOPO or pCR Blunt II (Life Technologies, Carlsbad, CA, USA) and sequenced. *Cis*-elements related to secondary cell wall formation were retrieved from the promoter 1500 bp upstream of the start codon with QIAGEN CLC Genomics Workbench (v20) and visualized online with IBS [69].

### 4.3. Phylogenetic Analysis

Full-length protein sequence alignment (MUSCLE) and generation of the phylogenetic tree using the approximate likelihood-ratio test (SH-like) in PhyML was performed online (phylogeny.fr, [70,71]).

### 4.4. Expression Profile Analysis by RT-qPCR and Histochemical GUS Staining

Total RNA was extracted from stems and roots of 4-month-old *P. tremula* × *P. alba* (*n* = 3, one tree per biological replicate) grown in the greenhouse using the Agilent Plant RNA isolation mini kit (Agilent, Santa Clara, CA, USA). Within the stem, three samples were harvested. The first one corresponded to the 2 first cm of the stem, including the apex and leaf primordia, corresponding to primary VT. For the second and third samples, a portion of the stem was removed at 50 cm below the apex (corresponding to secondary VT) and separated into bark (phloem) and wood (xylem). For the roots, primary VT corresponded to young root with primary structure, and secondary VT corresponded to roots with a diameter of 1 cm. The reverse transcription was carried out with GoScript Reverse Transcription Mix, Oligo(dT) kit (Promega, Madison, WI, USA). The qPCR was carried out with Luna Universal qPCR Master Mix and the LightCycler 480 system (Roche, Bâle, Switzerland) in technical triplicate. Amplification specificity was checked at the end of each qPCR run with a melt curve. The obtained values were normalized with two reference genes (*CYC063* and *CDC2* [72]). The gene relative expression was calculated using specific primers efficiency. Primers sequences are shown in Table S1.

Histochemical GUS staining was performed as described by Hemerly et al. [73] with modifications. After fixation in 90% acetone (1 h at −20 °C), samples were rinsed with PBS and incubated with GUS reaction buffer (50 mM PBS, 0.1% triton, 0.1 mM potassium ferricyanide, 0.1 mM potassium ferrocyanide, and 0.3 mg/mL 5-bromo-4-chloro-3-indolyl β-D-glucuronic acid (X-Gluc)). After GUS staining overnight, samples were decolored in 70% ethanol, and cross sections (60–100 µm thick) were made with an HM 650 V vibratome (Thermo Scientific, Waltham, MA, États-Unis, USA, http://www.thermoscientific.com) and observed under a light microscope.

### 4.5. Antibody Production, Immunoblotting and Immunolocalization

Polyclonal antibodies raised against specific peptides of UGT72AZ1 (TDNSLSKSQLLKQSPC), UGT72AZ2 (CKSVRFEDTLPAYLDR), and UGT72A2 (LTRPLKPLDSRSGEL) were produced and quality-checked externally (Eurogentec, Liège, Belgium). The cross-reactivity of the purified antibodies was tested by immunoblot with the recombinant proteins UGT72AZ1, AZ2, and A2 produced in *E. coli* BL21(DE3) strain in the vector pBAD-DEST49 (Invitrogen, Carlsbad, CA, États-Unis, USA) (Figure S7). The expression of the vector was induced with 0.02% arabinose (2 h at 37 °C). Proteins from induced culture were extracted from the crude extract and separated using an SDS-PAGE using a 12.5% acrylamide resolving gel with a Criterion Cell device (Bio-rad, Hercules, CA, USA). After the migration, proteins were transferred to PVDF Hybond-P membrane (Amersham Biosciences). The membrane was blocked with Odyssey blocking buffer (LI-COR Biosciences, Lincoln, NE, USA), then incubated overnight at 4 °C with primary antibody (at a dilution of 1/200 for anti-UGT72AZ1, 1/1000 for anti-UGT72AZ2 and 1/200 for anti-UGT72A2) in PBS buffer solution with 0.2% of Tween 20 (Sigma-Aldrich, Saint-Louis, MI, USA). Subsequently, it was washed thrice with TBST (Tris 1M pH8, NaCl 5M and 0.1% of Tween 20) and incubated with secondary antibodies (goat anti-rabbit immunoglobulin-G) diluted 1/10,000 in PBS buffer solution with 0.2% of Tween 20 at 37 °C during 1 h followed by 3 washes in TBST. Signals were revealed with *Odyssey Fc Imaging System* (LI-COR Biosciences). The image processing was realized with Image Studio Lite (LI-COR biosciences).

For immunolocalization, stem pieces were fixed with a solution of 4% formaldehyde and 0.1% Triton in 50 mM PIPES overnight at 4 °C with agitation after a vacuum for 30 min. After fixation, stem sections (100 µm) obtained using an HM 650 V vibratome were incubated with primary antibodies (diluted at 1/100 for anti-UGT72AZ1, 1/200 for anti-UGT72AZ2, and 1/100 for anti-UGT72A2 in 0.75x Odyssey blocking buffer + 0.2% DMSO) overnight at 4 °C. Sections were washed thrice with 50 mM PIPES buffer, incubated for 1 h 30 min at 37 °C with anti-rabbit antibodies conjugated to AlexaFluor 488 (1/500; Life Technologies) and washed thrice with 50 mM PIPES buffer before observation using a Zeiss confocal microscope LSM710. Secondary antibody was detected with the following parameters: excitation at 488 nm, emission recorded between 495 and 573 nm. Lignin autofluorescence was detected with the following parameters: excitation at 405 nm, emission recorded between 410 and 507 nm. All acquisition settings were similar for sections incubated with the different antibodies, and no change was made to image display post-acquisition.

### 4.6. Subcellular Localization of the poplar UGT72s Fused with GFP

Poplar *UGT72s* were cloned in the vector pK7FWG2 [74] by Gateway technology. *35S*::*AtWAK2–mCherry* construct was used as marker for ER [75]. Each construct was used to transform *A. tumefaciens* (strain C58RifPMP90) by electroporation. After separated preculture in LB medium with 10 mM MES buffer and 20 µM acetosyringone (supplemented with the selection antibiotic), bacterial suspensions were mixed, and agroinfiltration were performed in tobacco leaves. After 2 to 4 days, leaves were analyzed with a LSM710 confocal microscope. The wavelength of excitation for GFP is 488 nm, and the emission was recorded between 495 and 543 nm. The wavelength of excitation for mCherry is 543 nm, and the emission was recorded between 560 and 630 nm. The analysis was not performed for UGT72BB1 and UGT72B38.

### 4.7. Enzymatic Assay on Recombinant Proteins Produced in E. Coli

Recombinant protein production was performed in *E*. *coli* BL21(DE3)pLysS genotype using *UGT72*s cloned into pGEX-4T-1 (GE Healthcare, Chicago, IL, USA). Following transformation, a preculture grown at 37 °C was used to inoculate 500 mL of LB supplemented with ampicillin (100 µg/mL) and chloramphenicol (34 µg/mL). Induction with IPTG (final concentration of 1 mM) was performed at an OD_600_ of 0.5–0.6, followed by overnight incubation at 18 °C under agitation (200 rpm). Cells were then pelleted, washed, and stored at −80 °C until protein purification. The pellet was then resuspended in 10 mL GST-Wash/Bind buffer supplemented with PMSF (final concentration 1 mM) and lysozyme and ultrasonicated. The resulting bacterial lysate was centrifuged (30 min, 13,500 rpm at 4 °C), and the supernatant was applied to a column with GST•Bind™ resin (Novagen). GST-tagged proteins were allowed to bind to the resin overnight at 4 °C under soft agitation. After three washings, proteins were eluted in 5 consecutive fractions (300 µL each). The purity of these 5 fractions was investigated through SDS-PAGE, before pooling the pure fractions for quantification with the Roti-Nanoquant assay (Carl Roth).

The enzymatic assay was performed with 5 µg of purified protein and the following final concentrations: 100 mM Tris HCl pH7.5, 1 mM UDP-glucose, 600 µM substrate (coniferaldehyde, coniferyl alcohol, *p*-coumaraldehyde, sinapaldehyde, sinapyl alcohol, trans-cinnamic acid, *p*-coumaric acid, caffeic acid, ferulic acid, sinapic acid, and salicylic acid) in a total volume of 100 µL. Each reaction was performed in triplicate and incubated overnight at 30 °C. The reaction was stopped by heating at 75 °C for 10 min, centrifuged, and analyzed with a LC-UV-MS/MS system using the protocol described in Reference [76]. Identity of the glycosylated product was established by examination of the MS/MS spectra.

### 4.8. Generation of Transgenic Poplars Upregulating UGT72s, Expressing Promoter-GUS or UGT72A2-GFP Constructs

For the overexpression, *UGT72s* were cloned in PK7WG2D [74] and promoter-GUS constructs were obtained by inserting the different promoters into pKGWFS7 [74] by Gateway technology. The UGT72A2-GFP construct is the same as that used for agroinfiltration. The recombined plasmids were mobilized to *A. tumefaciens* (strain C58^Rif^PMP90). Poplar transformation was made as described in [77]. A minimum of 20 independent transgenic lines per construct were selected based on kanamycin resistance.

### 4.9. Lignin Content

Lignin content was determined in cell wall residue [78] made from wood and bark of stem and root of 4-month-old transgenic poplars and WT (at least in biological triplicate) using acetyl bromide digestion, as described previously [79].

### 4.10. Metabolite Extraction, HPLC-UV-MS and LC-MS Analyses

Plant material was ground in liquid nitrogen for each line (at least in biological triplicate). Metabolites were extracted on 200 mg (fresh weight) with 1 mL methanol 80% on ice over 4 h with several vortexing steps. After centrifugation, the extract was filtered on a 0.22 µm membrane, aliquoted, and 20 µL were injected in a HPLC-UV-MS system (Agilent 1260 Infinity II coupled to Advion Expression CMS L). Metabolites were separated using a water (0.1% formic acid)/acetonitrile gradient (10 to 30% ACN in 20 min) at 1 mL/minute on a C18 column (InfinityLab Poroshell 120 EC-C18, 100 × 4.6 mm i.d., 4 µm). Identification of coniferin and syringin was based on their retention time, absorption at 264 nm and mass spectra by comparison with authentic standards. Differences in phenolic abundance between WT and overexpressing lines were assessed by comparing UV profiles at 264 nm.

Accumulation of coniferin and syringin in *UGT72AZ1* and *UGT72AZ2* overexpressing lines was measured through LC-MS analysis on an ACQUITY UPLC I-Class system (Waters Corporation, Milford, MA, USA), coupled to a Synapt QTOF hybrid mass spectrometer (Waters Corporation). Chromatographic separation was carried out on an ACQUITY UPLC BEH C18 (150 × 2.1 mm, 1.7 μm) column from Waters, temperature was maintained at 40 °C. A 30 min gradient of two buffers was used: buffer A (99/1/0.1 H_2_O/ACN/formic acid pH3), buffer B (99/1/0.1 ACN/H_2_O/formic acid pH3); 95% A for 0.1 min decreased to 50% A in 30 min. The flow rate was set to 0.35 mL/minute, and the injection volume was 15 μL. The MS LockSpray ion source was operated in negative electrospray ionization (ESI) mode under the following specific conditions: capillary voltage: 2.5 kV; source temperature; 120 °C; desolvation gas temperature; 400 °C; desolvation gas flow; 550 L/h. Mass range was set from 100–1000 Da. Nitrogen (>99.5%) was employed as desolvation and cone gas. Leucin-enkephalin (250 pg/μL solubilized in water/ACN 1:1 (*v*/*v*), with 0.1% formic acid) was used for the lock mass calibration. Centroided data was recorded through Masslynx software (Waters). Chromatogram processing and data normalization was performed with Progenesis QI software v2.1 (Waters). Database maintenance for compound identification was done with JChem for Excel add-in (Chemaxon, Budapest, Hungary).

### 4.11. Accession Uumbers

Gene sequence data from this article are deposited in GenBank under the accession numbers MT181026 (*UGT72A2*), MT181027 (*UGT72AZ1*), MT181028 (*UGT72AZ2*), MT181029 (*UGT72B36*), MT181030 (*UGT72B37*), MT181031 (*UGT72B38*), MT181032 (*UGT72B39*), MT181033 (*UGT72BB1*), MT193261 (*pUGT72A2*), MT193262 (*pUGT72AZ1*), MT193263 (*pUGT72AZ2*), MT193264 (*pUGT72B36*), MT193265 (*pUGT72B37*), MT193266 (*pUGT72B38*), MT193267 (*pUGT72B39*), and MT193268 (*pUGT72BB1*).

The accession numbers of the Arabidopsis *UGT72s* are At3G50740 (*UGT72E1*), At5G26310 (*UGT72E2*), At5G66690 (*UGT72E3*), At4G01070 (*UGT72B1*), At1g01390 (*UGT72B2*), At1g01420 (*UGT72B3*), At4G36770 (*UGT72C1*), At2G18570 (*UGT72D1*), and At2G18560 (*UGT72D2P*). The *P. trichocarpa* accession numbers corresponding to the cloned UGT72s from *P*. *tremula* × *P*. *alba* are Potri.007G030300 (*UGT72AZ1*), Potri.007G030400 (*UGT72AZ2*), Potri.007G030500 (*UGT72A2*), Potri.014G041800 (*UGT72BB1*), Potri.014G096000 (*UGT72B36*), Potri.014G096100 (*UGT72B37*), Potri.003G138200 (*UGT72B38*), and Potri.002G168600 (*UGT72B39*).

## 5. Conclusions

The survey of the UGT72 family in poplar allowed the identification of members that are involved in the glycosylation of monolignols and that are clustered with Arabidopsis UGT72Es (group 1) and UGT72Bs (group 4). In addition, poplar *UGT72s*, like their Arabidopsis counterparts, are expressed within vascular tissues. No direct impact of the overexpression of poplar *UGT72s* on lignification was evidenced. Finally, the observed differences in subcellular localization suggest divergent functions of members of the poplar UGT72 family. Further studies on poplar mutants and identification of substrates are required to unravel the role of UGT72s, particularly in the lignification process or in defense towards pathogens in poplar.

## Figures and Tables

**Table 1:** Poplar UGT72s activity towards different phenylpropanoids.

Substrates	UGT72AZ2 (Group 1)	UGT72B37 (Group 4)	UGT72B39 (Group 4)
*p*-coumaraldehyde	−	+	−
coniferaldehyde	−	+	−
sinapaldehyde	−	+	−
coniferyl alcohol	−	+	+
sinapyl alcohol	−	+	−
*trans*-cinnamic acid	−	−	−
*p*-coumaric acid	−	−	−
caffeic acid	−	−	−
ferulic acid	+	−	−
sinapic acid	+	−	−
salicylic acid	−	−	−

− no activity detected; +, activity detected.

## Supplementary Materials

Supplementary materials can be found at https://www.mdpi.com/1422-0067/21/14/5018/s1.
