# Supplementary material for: Characterization of the UDP-glycosyltransferase UGT72 Family in Poplar and Identification of Genes Involved in the Glycosylation of Monolignols
Source: Int J Mol Sci. 2020 Jul 16;21(14):5018. doi: 10.3390/ijms21145018 (PMC7404001; doi:10.3390/ijms21145018)
Supplement: Supplementary file 1 [file ijms-21-05018-s001.zip › Figure S1.docx]

UGT72BB1 1 MAVVENETAKPHVAIMPSVGIGHITPLLEIAKRLVVLHDFHV-SFIVIAT-NEASAGQGSLLQASTL-PPGLDVVCLPTVDVFAVTTNGM
Potri_014G041900 1 --------------------MGQIIPLLEFSKRLVVDHDFHV-SFLVITTSNEASAAQDQLLQSPTF-PSGLDVVYLPPIDVFSVTTDDM
UGT72B36 1 ------MTQKPHLAFFPSPGMGHLIPLTELAKKFALNYNLSST--FIVPSIGPLPEAQKKVLGSL---PEGINYVSLPPVSFDDL-PG-I
UGT72B38 1 MDNIQGQEASPQVVIVPSPGMGHLIPFVELAKKLVHQHNFSVT--FIIPNDGSPMKSHRQLLQAL---PKGVSSVFLPPVNFDDL-PPDV
UGT72B37 1 ---MAETDSPPHVVILPSPGMGHLIPLIELAKRLVHQHNLSVT--FIIPTDGSPSKAQRSVLGAL---PSTIHSVFLPPVILSDL-PEDA
UGT72B39 1 ---MAQTDAPAHVAILPSPGMGHLIPLVELAKRLVHQHNFSIT--FVIPTDGSTSKAQRSVLGSL---PSAIHSVSLPPVNLSDL-PEDV
UGT72B1 1 ----MEESKTPHVAIIPSPGMGHLIPLVEFAKRLVHLHGLTVT--FVIAGEGPPSKAQRTVLDSL---PSSISSVFLPPVDLTDL-SSST
UGT72B3 1 ----MADGNTPHVAIIPSPGIGHLIPLVELAKRLLDNHGFTVT--FIIPGDSPPSKAQRSVLNSL---PSSIASVFLPPADLSDV-PSTA
UGT72B2 1 ----MAEANTPHIAIMPSPGMGHLIPFVELAKRLVQHDCFTVT--MIISGETSPSKAQRSVLNSL---PSSIASVFLPPADLSDV-PSTA
UGT72C1 1 --------MELHGALVASPGMGHAVPILELGKHLLNHHGFDRVTVFLVTDDVSRS--KSLIG-KTLMEEDPKFVIRFIPLDVSGQ-DLSG
UGT72D1 1 -------MDQPHALLVASPGLGHLIPILELGNRLSSVLNIHV-TILAVTSGSSSP--TETEAIHAAAARTICQITEIPSVDVDNLVEPDA
UGT72D2P 1 -------MEHPHVLLVASPGLGHLIPALELGNRLSFVLNVHV-TILAITSGSSSL--TETETIHTAAARGTCEIIELPSVDIDHLVEPDA
UGT72E1 1 ---M--KITKPHVAMFASPGMGHIIPVIELGKRLAGSHGFDV-TIFVLETDAASA--QSQFLNSPGCDAALVDIVGLPTPDISGLVDPSA
UGT72E3 1 ---M--HITKPHAAMFSSPGMGHVLPVIELAKRLSANHGFHV-TVFVLETDAASV--QSKLLNSTGV-----DIVNLPSPDISGLVDPNA
UGT72E2 1 ---M--HITKPHAAMFSSPGMGHVIPVIELGKRLSANNGFHV-TVFVLETDAASA--QSKFLNSTGV-----DIVKLPSPDIYGLVDPDD
UGT72A2 1 MENT--TGSKPHLVLLASLGIGHLIPILELGKRLFTHHNFDI-TIFVAVSHSSAA--ESQVLQSAMT-PKLCEIVELPTVNISRLVSPEA
UGT72AZ2 1 ---M--QNTKPHAALLASPGMGHLIPVLELGKRLVTYHGFHV-TLFVVATDASTT--QSLLK--EPY-PN-INIITLPLVDISGLIDPAA
UGT72AZ1 1 ---M--QNTKPHAALLSSPGMGHLIPVLELGKRLVTNHGFTV-TIFVVTTDNSLS--KSQLLKQSPC-PDLLSIVLLPPVDVSSLITPTT
Potri.007G029800 1 ---M--QNTKPHAALLSSPGMGHLIPVLELGKCLVTNHGFTV-TIFVVTTDNSLS--KSQLLKQSPC-PDLLNIVLLPPVDVSSLITPTT

*

UGT72BB1 88 PVAARLCAIVQEAIKSLKAVLVEL----GKIKAVVVDLFCTQAFDICSELSI-PAYLFFTASIALLNFSLYLPTLDREVEGEFVDLPEPV
Potri_014G041900 69 LMLTRLCVMVEESLKSLKSVLKEL----GELRAVVIDKFFTQAFDVCCELSI-PAYLFYTSAIVMLTFSLSLPTLDCEVEGEFVDLAEPL
UGT72B36 78 RAETQISLTITRSLSSVRDVLKSLAAS-TRLVALVLDLFGTDVIDIASELSV-PSYIASLSTGMTLSLHFYLPKLDQMVSCEYRDLPEPV
UGT72B38 85 LVETRITLSLTRSLDALRDTLKTLTDS-TKVVALVVDLFGPFAFEIAKEFDV-LPFVFFPTNVMLLSLSFHLPRLDETYSGEYKDMTEPV
UGT72B37 82 KIETLISLTVARSLPSLRDALSSLVASGTRVVALVVDLFGTDAFDVAREFKA-SPYIFFPAPAMALSLFFYLPKLDEMVSCEYSEMQEPV
UGT72B39 82 KIETTISLTVARSLPSLRDVFRSLVDGGARVVALVVDLFGTDAFDVAREFNV-SPYIFFPSTAMALSLFFYLPKLDEMVSCEYREMQEPV
UGT72B1 81 RIESRISLTVTRSNPELRKVFDSFVEGGRLPTALVVDLFGTDAFDVAVEFHV-PPYIFYPTTANVLSFFLHLPKLDETVSCEFRELTEPL
UGT72B3 81 RIETRISLTVTRSNPALRELFGSLSAEKRLPAVLVVDLFGTDAFDVAAEFHV-SPYIFYASNANVLTFLLHLPKLDETVSCEFRELTEPV
UGT72B2 81 RIETRAMLTMTRSNPALRELFGSLSTKKSLPAVLVVDMFGADAFDVAVDFHV-SPYIFYASNANVLSFFLHLPKLDKTVSCEFRYLTEPL
UGT72C1 79 SLLTKLAEMMRKALPEIKSSVMEL---EPRPRVFVVDLLGTEALEVAKELGIMRKHVLVTTSAWFLAFTVYMASLDKQELYKQLSSIGAL
UGT72D1 81 TIFTKMVVKMRAMKPAVRDAVKLM---KRKPTVMIVDFLGTELMSVADDVGMTAKYVYVPTHAWFLAVMVYLPVLDTVVEGEYVDIKEPL
UGT72D2P 81 TVVTXIVSKMREMKSTVRDAVKSM---KQKPTVMIVDFFGTALLSIT-DVGVTSKYVYIPSHAWFLALIVYLPVLDKVMEGEYVDIKEPM
UGT72E1 83 FFGIKLLVMMRETIPTIRSKIEEM---QHKPTALIVDLFGLDAIPLGGEFNM-LTYIFIASNARFLAVALFFPTLDKDMEEEHIIKKQPM
UGT72E3 78 HVVTKIGVIMREAVPTLRSKIVAM---HQNPTALIIDLFGTDALCLAAELNM-LTYVFIASNARYLGVSIYYPTLDEVIKEEHTVQRKPL
UGT72E2 78 HVVTKIGVIMRAAVPALRSKIAAM---HQKPTALIVDLFGTDALCLAKEFNM-LSYVFIPTNARFLGVSIYYPNLDKDIKEEHTVQRNPL
UGT72A2 85 AVATQICVVMREIKPALRSAISAL---SFRPAALIADLFGSEAMMVADEFEM-PRYVYVPSNAWFLALTIYVPILDEAVQGEYLDQKEPL
UGT72AZ2 79 TVVTKLAVMMRETLPSLRSAILAL---KSPPTALIVDLFGTKAFAVAEEFNM-LKYVFDTSNAWFFAITIYVPTIDRNLEDKHIIQKQPL
UGT72AZ1 82 GILAQLAIMMREALPKLRSAILAM---KFCPTVLIVDFFGTEAMVIADEFNM-LKYAFMTSTAWFLALTLHMPTIDKAIEDDHVKNQQAL
Potri.007G029800 82 GILAQLAIMMRKALPKLRSAILAM---EFCPTVLIVDFFGTEAMVIADEFNM-LKYAFMTSTAWFLALTLHMPAIDKAIEDNHVKNQQAL

*

UGT72BB1 173 KVPGC-PPIRPEDLLDQVKNRKIDEYKWYLFHSSRFHLGAGIFLNSWEGLEPANFKAITEDPFFKQIH-TPPVHPVGPLIKLEEPLT--A
Potri_014G041900 154 KVPGC-PPFPIEDLFDPLKNRKIDEYKWLLFHSSRFHLAAGIFVNSWKELESVTYKAITEDPFFKQIP-TPPVLPVGPLIKGEEPLT--A
UGT72B36 166 LLPGCGIAVHGRDLPDPIQDRKDDAYKWFLHHSKRHSLAEGILLNSFVDLEPETIKALQDQELGN----LPPIYPVGPIIYSGSSMGA--
UGT72B38 173 RLPGC-VPVQGRDLVDPAQDRKGDAYKWILHICKLYNSAAGIMVNSFIDLEPGAFKALMEENNIG----KPPIFPVGPLTQTGSTSGDV-
UGT72B37 171 EIPGC-LPIHGGELLDPTRDRKNDAYKWLIYHTKRYRLAEGVMVNSFIDLERGALKALQE-EGPG----KPPVYPVGPLVNMGSNTSGV-
UGT72B39 171 KIPGC-LPIHGGELLDPTQDRKNDAYKWLLYHTKRYRLAEGVMVNSFMDLEKGALKALQE-VEPG----KPTVYPVGPLVNMDSR-AAV-
UGT72B1 170 MLPGC-VPVAGKDFLDPAQDRKDDAYKWLLHNTKRYKEAEGILVNTFFELEPNAIKALQE-PGLD----KPPVYPVGPLVNIGKQEAKQT
UGT72B3 170 IIPGC-VPITGKDFVDPCQDRKDESYKWLLHNVKRFKEAEGILVNSFVDLEPNTIKIVQE-PAPD----KPPVYLIGPLVNSGSHDADVN
UGT72B2 170 KIPGC-VPITGKDFLDTVQDRNDDAYKLLLHNTKRYKEAKGILVNSFVDLESNAIKALQE-PAPD----KPTVYPIGPLVNTSSSNVNLE
UGT72C1 166 LIPGC-SPVKFERAQDPRKY--IRELAESQRIGDEVITADGVFVNTWHSLEQVTIGSFLDPENLGRVMRGVPVYPVGPLVRPAEPGL--K
UGT72D1 168 KIPGC-KPVGPKELMETMLDRSGQQYKECVRAGLEVPMSDGVLVNTWEELQGNTLAALREDEELSRVM-KVPVYPIGPIVRTNQHVD--K
UGT72D2P 167 KIPGC-KPVGPKELLDTMLDRSDQQYRDCVQIGLEIPMSDGVLVNTWGELQGKTLAALREDIDLNRVI-KVPVYPIGPIVRTNVLIE--K
UGT72E1 169 VMPGC-EPVRFEDTLETFLDPNSQLYREFVPFGSVFPTCDGIIVNTWDDMEPKTLKSLQDPKLLGRIA-GVPVYPIGPLSRPVDPSK--T
UGT72E3 164 TIPGC-EPVRFEDIMDAYLVPDEPVYHDLVRHCLAYPKADGILVNTWEEMEPKSLKSLQDPKLLGRVA-RVPVYPVGPLCRPIQSST--T
UGT72E2 164 AIPGC-EPVRFEDTLDAYLVPDEPVYRDFVRHGLAYPKADGILVNTWEEMEPKSLKSLLNPKLLGRVA-RVPVYPIGPLCRPIQSSE--T
UGT72A2 171 KIPGC-KAVQPEDVVDPMLDRTDQQYLECVRMGMEIPKCDGILLNIWEDLEPKTLEALRDEELLGQLC-KAPVYPVGPLTRPLKPLD--S
UGT72AZ2 165 RIPGC-KSVRFEDTLPAYLDRNDQTYIEYKRIGIEMPMADGILMNTWEDLEPTTLGALRDFQMLGRVA-KAPVYPIGPLARPVGPSV--P
UGT72AZ1 168 LIPGC-KSLEFRDTFEPVLDRNDQMYMEYKRMGVEMQKFDGILVNTWQDLEGTTLGALEDQKRLGRVA-QVPIYPVGPLVRAITPG---P
Potri.007G029800 168 LIPGC-KSLEFRDTFEPVLDRNDQMYIEYKRMGVEMQKFDGILVNTWQDLEGTTLGALEDEKRLGRVA-QVPIYPVGPLVRAITPG---P


UGT72BB1 259 SDADCLAWLDKQPPNSVLFVSLGSGGTLTAEQLTELAWGLELSHQRFIFVVRKPANSSASAAFFNAGSD--VNDPRTYLPTGFLERTQER
Potri_014G041900 240 RDIEYLAWLDKQPSDSVLFVALGSGGTLTADQLTELAWGIELSHQRFVFVARKPTNSSASAAVFTAGSD--IGNPVLKCVNS-------R
UGT72B36 250 SGHECLQWMDDQPNGSVLYISFGSGGTLSFEQLNELAMGLEISEQKFLWVVRSPDK-SASASYFSATSN---TDPYSFLPKGFLDRTKGQ
UGT72B38 257 GESECLNWLDKQPKGSVLFVSFGSGGTLSHAQLNELSLGLEMSGQRFPWVVRSPHDEAANATYFGIRSS---DDPLAFLPEGFLDRTKGV
UGT72B37 254 EGSECLKWLDDQPLGSVLFVSFGSGGTLSLDQIAELALGLEMSEQRFLWVARVPNDKVANATYFSVDNH---KDPFDFLPKGFLDRTKGR
UGT72B39 253 EGSECLKWLDDQPHGSVLFVSFGSGGTLSLDQITELALGLEMSEQRFLWVVRSPNDEVSNATFFSVDSH---KDPFDFLPKGFSDRTKGR
UGT72B1 254 EESECLKWLDNQPLGSVLYVSFGSGGTLTCEQLNELALGLADSEQRFLWVIRSPSG-IANSSYFDSHSQ---TDPLTFLPPGFLERTKKR
UGT72B3 254 DEYKCLNWLDNQPFGSVLYVSFGSGGTLTFEQFIELALGLAESGKRFLWVIRSPSG-IASSSYFNPQSR---NDPFSFLPQGFLDRTKEK
UGT72B2 254 DKFGCLSWLDNQPFGSVLYISFGSGGTLTCEQFNELAIGLAESGKRFIWVIRSPSE-IVSSSYFNPHSE---TDPFSFLPIGFLDRTKEK
UGT72C1 251 --HGVLDWLDLQPKESVVYVLLGVVGALTFEQTNELAYGLELTGHRFVWVVRPPAEDDPSASMFDKTKN--ETEPLDFLPNGFLDRTKDI
UGT72D1 254 P-NSIFEWLDEQRERSVVFVCLGSGGTLTFEQTVELALGLELSGQRFVWVLRRPAS------YLGAISS-DDEQVSASLPEGFLDRTRGV
UGT72D2P 253 P-NSTFEWLDKQEERSVVYVCLGSGGTLSFEQTMELAWGLELSCQSFLWVLRKPPS------YLGASSK-DDDQVSDGLPEGFLDRTRGV
UGT72E1 255 N-HPVLDWLNKQPDESVLYISFGSGGSLSAKQLTELAWGLEMSQQRFVWVVRPPVDGSACSAYLSANSGKIRDGTPDYLPEGFVSRTHER
UGT72E3 250 D-HPVFDWLNKQPNESVLYISFGSGGSLTAQQLTELAWGLEESQQRFIWVVRPPVDGSSCSDYFSAKGGVTKDNTPEYLPEGFVTRTCDR
UGT72E2 250 D-HPVLDWLNEQPNESVLYISFGSGGCLSAKQLTELAWGLEQSQQRFVWVVRPPVDGSCCSEYVSANGGGTEDNTPEYLPEGFVSRTSDR
UGT72A2 257 RSGELFLWLDKRPSESVIYVSFGSGGALSLEQMVELAWGLELSQQRFIWVVRSPSRKTGDGSFFSAGSG-EANSMASCFPEGFLDRIQEV
UGT72AZ2 251 R-NQVLNWLDNQPYESVIYVSFGSGGTLSSEQMAELAWGLELSKQRFVWVVRPPVDNDADGAFFNLDDG-S-EGIPSFLPEGFLDRTSEV
UGT72AZ1 253 K-SEMLEWLDMQPVESVIYVSFGSGGALSAKQTTELACGLESSGQRFIWVVRPPIEGDSAATVFKTNHR-T-DDTPDFLPDGFLTRTRKT
Potri.007G029800 253 K-SEMLEWLDMQPIESVIYVSFGSGGALSARQTTELACGLESSGQRFIWVVRPPIEGDSAATVFKTKHR-T-DDTPDFLPDGFLTRTRKM

UGT72BB1 347 GLVVPSWATQVLVLRHPSTGGFLTHCGWNSTLEAVTHGIPMIAWPLYAEQRMNATILAEEIGIAIKPAAEPGASLVGREEVKRVVRLAIL
Potri_014G041900 321 D-------NPVLF-----------FSLSARVAQTIINGVPLIAWPLFAEQRMNATILAEQVGIAVKPVVKPGESLVGREEVERVVRLVIE
UGT72B36 336 GLVVPSWAPQIQVLSHGSTGGFLTHCGWNSTLESIVHGVPLIAWPLYAEQKTNAVLLSAGLKVALRPEVD-GNGLVGREEIAKVVKGLMQ
UGT72B38 344 GLVVPSWAPQIQVLSHSSTGGFLTHCGWNSILESVVNGVPLIAWPLYAEQRMNSVLLADGLKVALRVKVN-ENGLVMKEDIANYARSIFE
UGT72B37 341 GLVVPSWAPQAQVLSHGSTGGFLTHCGWNSTLESVVNGVPLIVWPLYAEQKMNAWMLTKDIKVALRPKAS-ENGLFEREEIANVVRGLME
UGT72B39 340 GLAVPSWAPQPQVLSHGSTGGFLTHCGWNSTLESVVNGVPLIVWPLYAEQKMNAWMLTKDIKVALRPKAS-ENGLVGRKEIANAVRGLME
UGT72B1 340 GFVIPFWAPQAQVLAHPSTGGFLTHCGWNSTLESVVSGIPLIAWPLYAEQKMNAVLLSEDIRAALRPRAG-DDGLVRREEVARVVKGLME
UGT72B3 340 GLVVGSWAPQAQILTHTSIGGFLTHCGWNSSLESIVNGVPLIAWPLYAEQKMNALLLV-DVGAALRARLG-EDGVVGREEVARVVKGLIE
UGT72B2 340 GLVVPSWAPQVQILAHPSTCGFLTHCGWNSTLESIVNGVPLIAWPLFAEQKMNTLLLVEDVGAALRIHAG-EDGIVRREEVVRVVKALME
UGT72C1 337 GLVVRTWAPQEEILAHKSTGGFVTHCGWNSVLESIVNGVPMVAWPLYSEQKMNARMVSGELKIALQINVA--DGIVKKEVIAEMVKRVMD
UGT72D1 336 GIVVTQWAPQVEILSHRSIGGFLSHCGWSSALESLTKGVPIIAWPLYAEQWMNATLLTEEIGVAVRTSELPSERVIGREEVASLVRKIMA
UGT72D2P 335 GLVVTQWAPQVEILSHRSIGGFLSHCGWSSVLESLTKGVPIIAWPLYAEQWMNATLLTEEIGMAIRTSELPSKKVISREEVASLVKKIVA
UGT72E1 344 GFMVSSWAPQAEILAHQAVGGFLTHCGWNSILESVVGGVPMIAWPLFAEQMMNATLLNEELGVAVRSKKLPSEGVITRAEIEALVRKIMV
UGT72E3 339 GFMIPSWAPQAEILAHQAVGGFLTHCGWSSTLESVLCGVPMIAWPLFAEQNMNAALLSDELGISVRVDDP--KEAISRSKIEAMVRKVMA
UGT72E2 339 GFVVPSWAPQAEILSHRAVGGFLTHCGWSSTLESVVGGVPMIAWPLFAEQNMNAALLSDELGIAVRLDDP--KEDISRWKIEALVRKVMT
UGT72A2 346 GLVIQDWAPQVDILNHPSVGGFISHCGWNSTLESITNGVPLIAWPLYAEQRMNAALLTEELGVAVRPNILASDGMVGREEVEMMIRKITV
UGT72AZ2 338 GLAVPMWAPQVEILAHPSVGGFLSHCGWNSTLESIANGVPMIAWPLYAEQKMNATILTEELGVAVQPKTLASERVVVRAEIETMVRKIME
UGT72AZ1 340 GLVVPMWAPQTEILNHPAVGGFVSHCGWNSTLESIVNGVPMITWPLFAEQGMNAAMLTEDIGVAIRSKSLPAKEVVGRGEIETMVRTIM-
Potri.007G029800 340 GLVVPMWAPQTEILSHPSVGGFVSHGGWNSTLESIVNGVPMITWPLYAEQGMNAAMLTEDIGVAIRSKSLPAKEVVVREEIETMVRTIM-
 #

UGT72BB1 437 ----EGKEMRKKIEELKDSAAKAMEIG--GSSYDSLACLAKEWKS---------------
Potri_014G041900 393 GE--KGRDMRRRTGELKESAAKALEIG--GSSHDALERVAKEWKAESNV-----------
UGT72B36 425 GE--EGAAIRSRMKGLKEAAAKAVSEE--GSSTKSLHELVSKWKN---------------
UGT72B38 433 GE--EGKSIKSKMNELKSAATRALSED--GSSTKSLAEVARIWKDHKK------------
UGT72B37 430 GE--EGKRVRNRMKDLKDAAAGVLSED--GPSTKALSEVARKWKNHKCTQDCN-------
UGT72B39 429 GE--EGKRVRNRMKDLKEAAARVLSED--GS----LSEVAHRWNNQTCT-----------
UGT72B1 429 GE--EGKGVRNKMKELKEAACRVLKDD--GTSTKALSLVALKWKAHKKELEQNGNH----
UGT72B3 428 GE--EGNAVRKKMKELKEGSVRVLRDD--GFSTKSLNEVSLKWKAHQRKIDQEQESFL--
UGT72B2 429 GE--EGKAIGNKVKELKEGVVRVLGDD--GLSSKSFGEVLLKWKTHQRDINQETSH----
UGT72C1 425 EE--EGKEMRKNVKELKKTAEEALNMTHIPSAYFT-------------------------
UGT72D1 426 EEDEEGQKIRAKAEEVRVSSERAW-SK-DGSSYNSLFEWAKRCYLVP-------------
UGT72D2P 425 EEDKEGRKIKTKAEEVRVSSERAW-TH-GGSSHSSLFEWAKRCGLVS*------------
UGT72E1 434 EE--EGAEMRKKIKKLKETAAESLSCDG-GVAHESLSRIADESEHLLERVRCMARGA---
UGT72E3 427 ED--EGEEMRRKVKKLRDTAEMSLSIHGGGSAHESLCRVTKECQRFLECVGDLGRGA---
UGT72E2 427 EK--EGEAMRRKVKKLRDSAEMSLSIDGGGLAHESLCRVTKECQRFLERVVDLSRGA---
UGT72A2 436 DK--EATNIRNRVKKLKHSAAETV-RKG-GSSHNALSLVAKQCELSWKSMEVKASWNA--
UGT72AZ2 428 DE--EGFGIRKRVNELKHSGEKALSSKG-GSSYNSLSQIAKQCELSLHFQKAKAQGA---
UGT72AZ1 429 DK--G-DARRARAKTLKSSAEKAL-SNG-GSSYNSLAHVANDCETAFEYLKAKAQGA---
Potri.007G029800 429 DK--G-DARRARAKTLKSSAEKAL-SKG-GSSYNSLAHVANDCETAFKYLKVKATWTST-
 ●●●

**Figure S1.** Multiple sequence alignment of UGT72 from poplar and Arabidopsis. The sequences were aligned using Clustal Omega (Madeira et al., 2019) and visualized using BoxShade (https://embnet.vital-it.ch/software/BOX_form.html). The PSPG domain is marked by a box. The histidine (H) in the N-terminal domain was identified as a key residue for enzyme *O*-glycosylation activity and aspartic acid (D) for enzyme catalysis (Shao et al., 2005; Offen et al., 2006; Brazier-Hicks et al., 2007). The glutamine (Q) in the C-terminal domain is necessary for UDP-glucose recognition and specificity (Kubo et al., 2004). The C-terminal GSS sequence is a special feature of monoglucoside UGT (Huang et al., 2018). *, catalytically active amino acids; #, UDP-glucose recognition specificity; ●●●, GSS sequence. Residues highlighted in black are identical and residues highlighted in grey have similar physicochemical properties. Arabidopsis accession numbers are At3G50740 (UGT72E1), At5G26310 (UGT72E2), At5G66690 (UGT72E3), At4G01070 (UGT72B1), At1g01390 (UGT72B2), At1g01420 (UGT72B3), At4G36770 (UGT72C1), At2G18570 (UGT72D1) and At2G18560 (UGT72D2P). *P. tremula x P. alba* accession numbers are MT181026 (UGT72A2), MT181027 (UGT72AZ1), MT181028 (UGT72AZ2), MT181029 (UGT72B36), MT181030 (UGT72B37), MT181031 (UGT72B38), MT181032 (UGT72B39) and MT181033 (UGT72BB1).

**References:** Madeira F, Park YM, Lee J, et al. The EMBL-EBI search and sequence analysis tools APIs in 2019. Nucleic Acids Research. 2019 Jul;47(W1):W636-W641. DOI: 10.1093/nar/gkz268.
