# Supplementary material for: Characterization of the UDP-glycosyltransferase UGT72 Family in Poplar and Identification of Genes Involved in the Glycosylation of Monolignols
Source: Int J Mol Sci. 2020 Jul 16;21(14):5018. doi: 10.3390/ijms21145018 (PMC7404001; doi:10.3390/ijms21145018)
Supplement: Supplementary file 1 [file ijms-21-05018-s001.zip › Figure S2.pptx]

## Slide 1
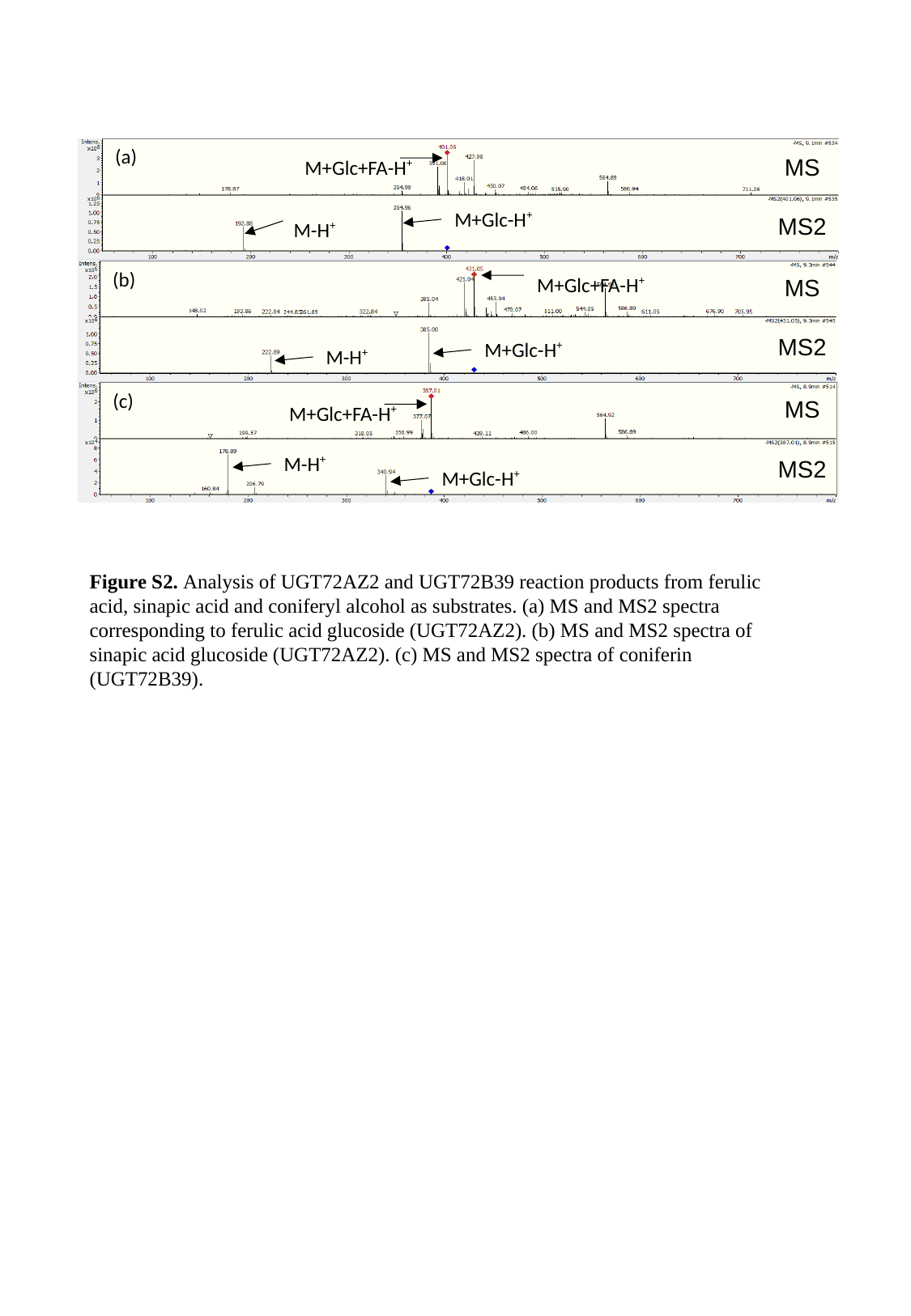

(a)
M+Glc+FA-H+
M+Glc-H+
M-H+
M+Glc+FA-H+
M+Glc-H+
M-H+
(b)
M+Glc+FA-H+
M-H+
M+Glc-H+
(c)
MS
MS2
MS
MS2
MS
MS2
Figure S2. Analysis of UGT72AZ2 and UGT72B39 reaction products from ferulic acid, sinapic acid and coniferyl alcohol as substrates. (a) MS and MS2 spectra corresponding to ferulic acid glucoside (UGT72AZ2). (b) MS and MS2 spectra of sinapic acid glucoside (UGT72AZ2). (c) MS and MS2 spectra of coniferin (UGT72B39).
