# Supplementary material for: Characterization of the UDP-glycosyltransferase UGT72 Family in Poplar and Identification of Genes Involved in the Glycosylation of Monolignols
Source: Int J Mol Sci. 2020 Jul 16;21(14):5018. doi: 10.3390/ijms21145018 (PMC7404001; doi:10.3390/ijms21145018)
Supplement: Supplementary file 1 [file ijms-21-05018-s001.zip › Figure S3.pptx]

## Slide 1
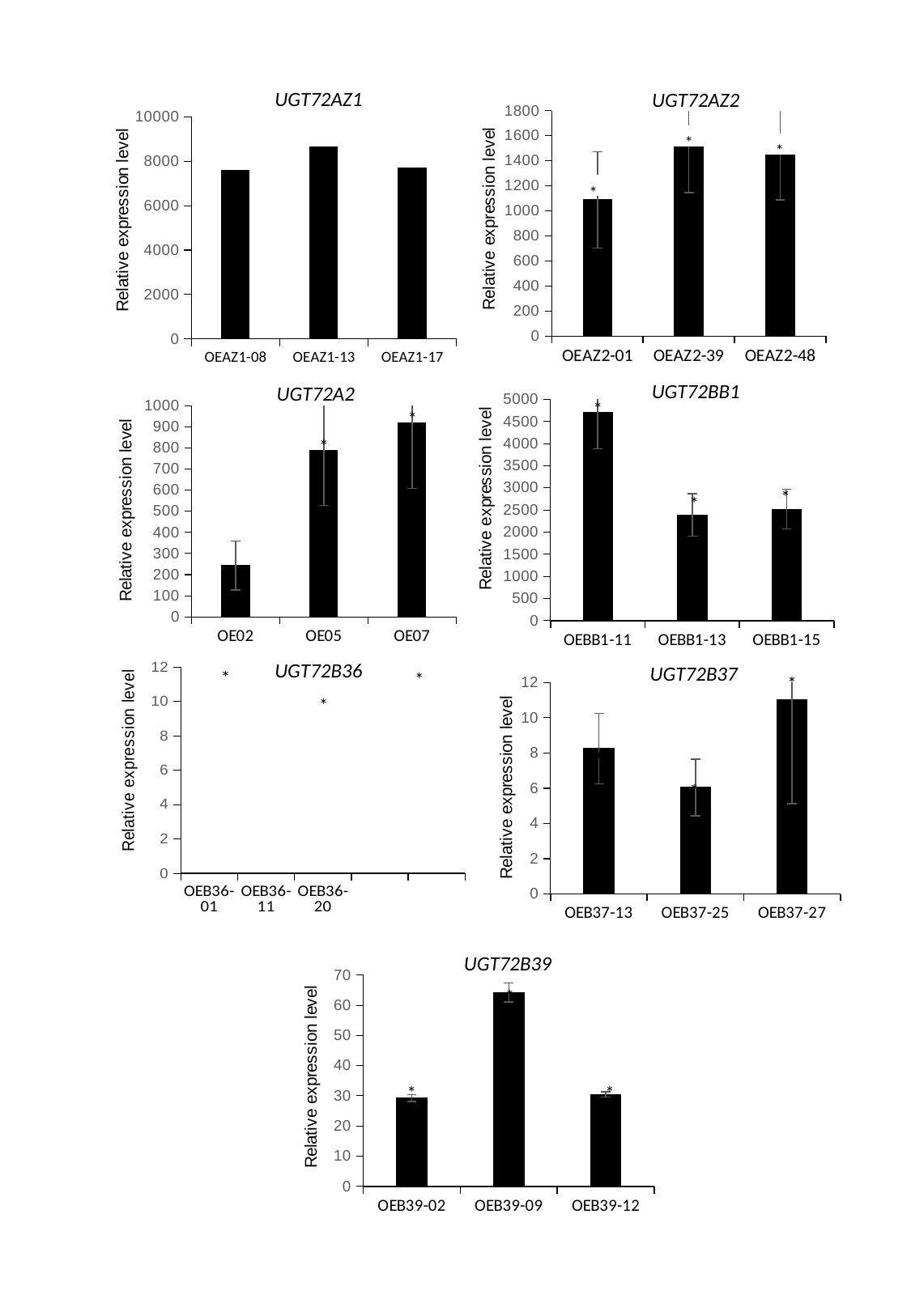

UGT72AZ1
UGT72AZ2
### Chart
| Category | |
|---|---|
| OEAZ1-08 | 7574.263806386676 |
| OEAZ1-13 | 8658.14104862599 |
| OEAZ1-17 | 7703.750806301686 |
### Chart
| Category | Niveau de surexpression (moy 3 réplicas) |
|---|---|
| OEAZ2-01 | 1088.2630180887936 |
| OEAZ2-39 | 1591.3158225099312 |
| OEAZ2-48 | 1503.0203683229254 |*
*
*
UGT72BB1
UGT72A2
### Chart
| Category | |
|---|---|
| OEBB1-11 | 4703.419137237132 |
| OEBB1-13 | 2385.5788753879615 |
| OEBB1-15 | 2518.2344983034964 |*
*
*
### Chart
| Category | |
|---|---|
| OE02 | 242.65950177455832 |
| OE05 | 789.0087439023616 |
| OE07 | 917.925085273232 |*
*
### Chart
| Category | Overexpression level |
|---|---|
| OEB36-01 | 20.24312749324734 |
| OEB36-11 | 19.62813133162123 |
| OEB36-20 | 22.4615663429245 |*
*
*
UGT72B36
UGT72B37
### Chart
| Category | Expression |
|---|---|
| OEB37-13 | 8.25337041811746 |
| OEB37-25 | 6.04475396588477 |
| OEB37-27 | 11.01962835722623 |*
*
*
UGT72B39
### Chart
| Category | Expression |
|---|---|
| OEB39-02 | 29.22512645868571 |
| OEB39-09 | 64.23738730881773 |
| OEB39-12 | 30.448841727996026 |*
*
*

## Slide 2
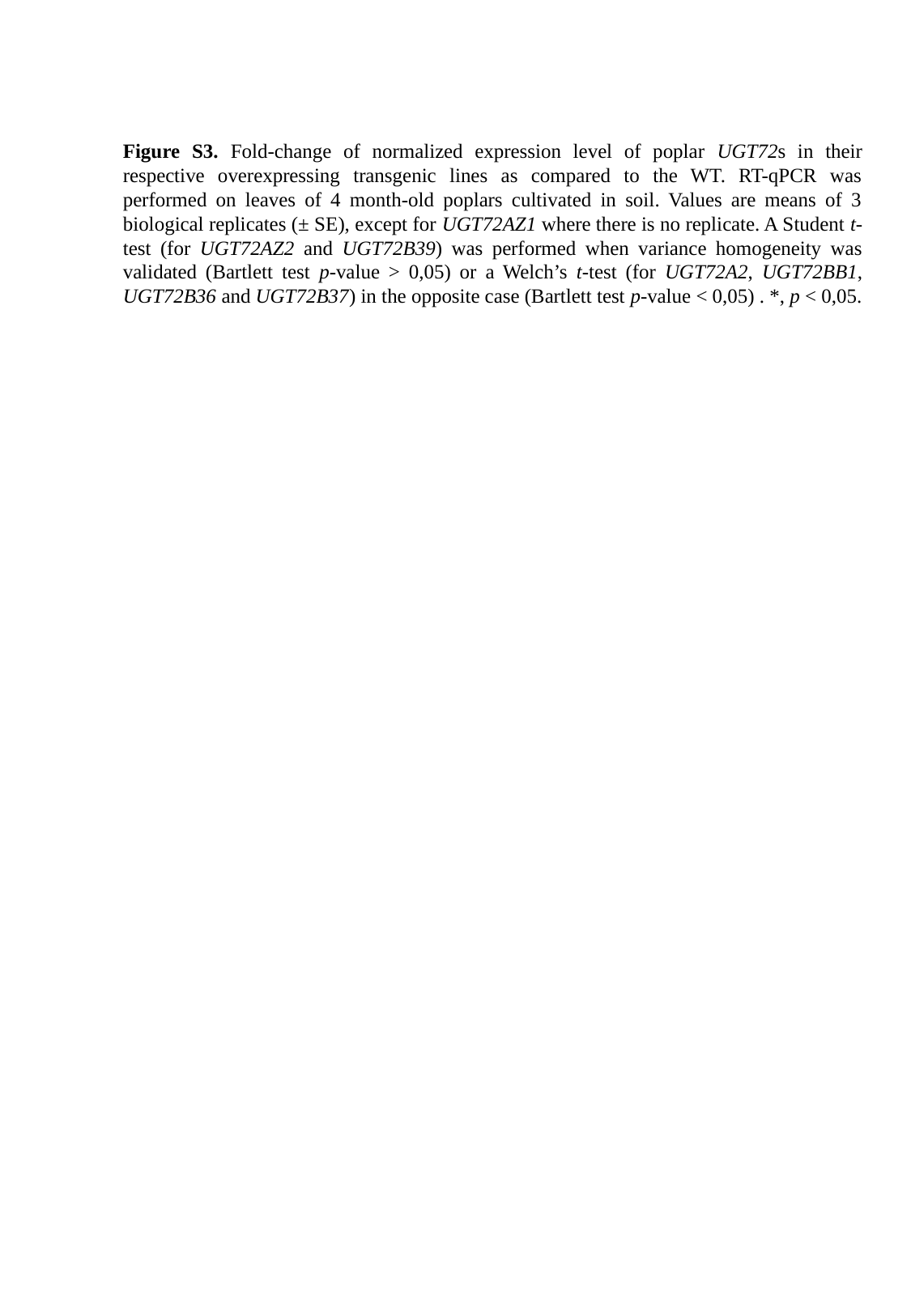

Figure S3. Fold-change of normalized expression level of poplar UGT72s in their respective overexpressing transgenic lines as compared to the WT. RT-qPCR was performed on leaves of 4 month-old poplars cultivated in soil. Values are means of 3 biological replicates (± SE), except for UGT72AZ1 where there is no replicate. A Student t-test (for UGT72AZ2 and UGT72B39) was performed when variance homogeneity was validated (Bartlett test p-value > 0,05) or a Welch’s t-test (for UGT72A2, UGT72BB1, UGT72B36 and UGT72B37) in the opposite case (Bartlett test p-value < 0,05) . *, p < 0,05.
