# Supplementary material for: Characterization of the UDP-glycosyltransferase UGT72 Family in Poplar and Identification of Genes Involved in the Glycosylation of Monolignols
Source: Int J Mol Sci. 2020 Jul 16;21(14):5018. doi: 10.3390/ijms21145018 (PMC7404001; doi:10.3390/ijms21145018)
Supplement: Supplementary file 1 [file ijms-21-05018-s001.zip › Figure S4.pptx]

## Slide 1
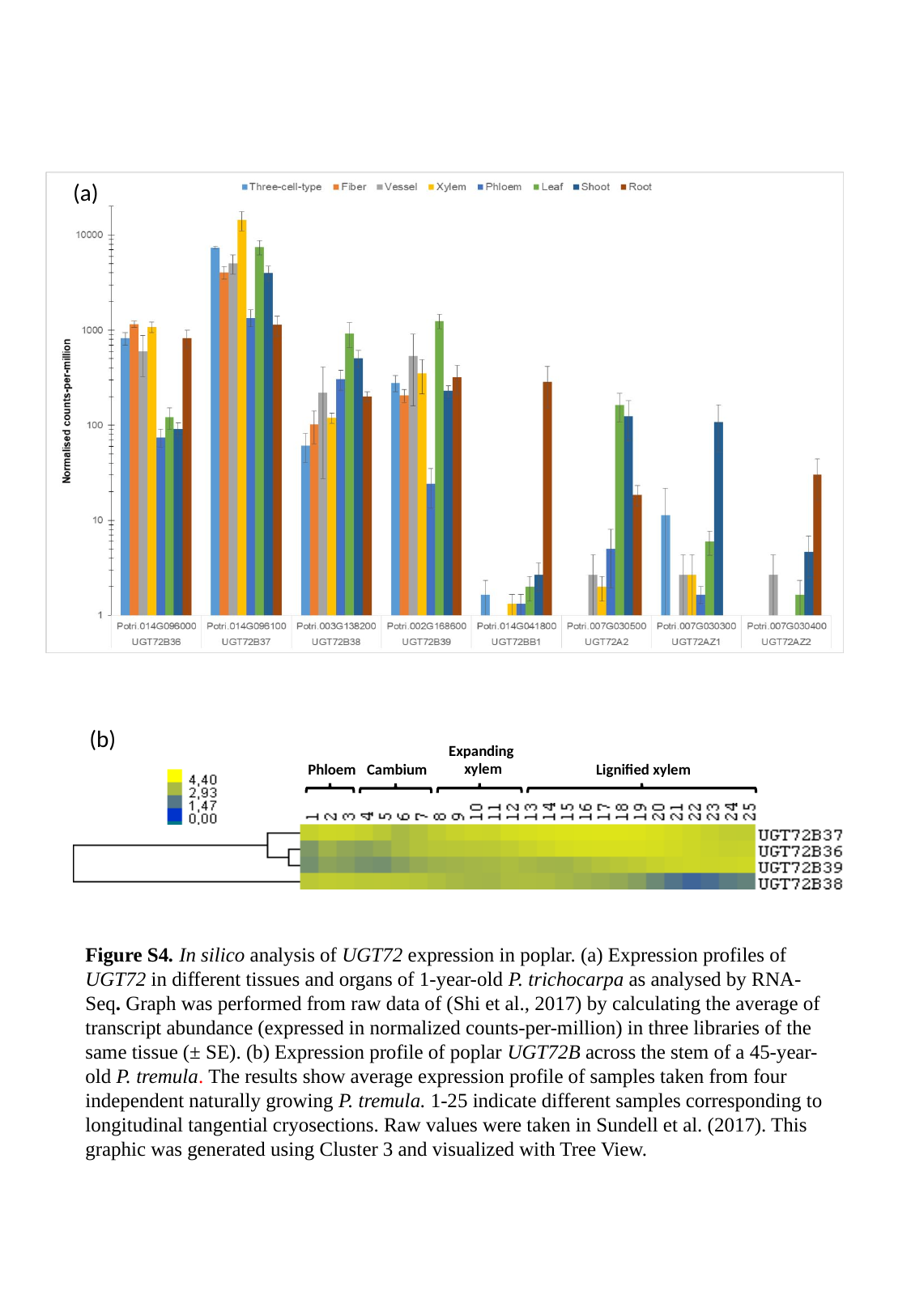

(a)
(b)
Expanding
 xylem
Phloem
Cambium
Lignified xylem
Figure S4. In silico analysis of UGT72 expression in poplar. (a) Expression profiles of UGT72 in different tissues and organs of 1-year-old P. trichocarpa as analysed by RNA-Seq. Graph was performed from raw data of (Shi et al., 2017) by calculating the average of transcript abundance (expressed in normalized counts-per-million) in three libraries of the same tissue (± SE). (b) Expression profile of poplar UGT72B across the stem of a 45-year-old P. tremula. The results show average expression profile of samples taken from four independent naturally growing P. tremula. 1-25 indicate different samples corresponding to longitudinal tangential cryosections. Raw values were taken in Sundell et al. (2017). This graphic was generated using Cluster 3 and visualized with Tree View.
