# Supplementary material for: Characterization of the UDP-glycosyltransferase UGT72 Family in Poplar and Identification of Genes Involved in the Glycosylation of Monolignols
Source: Int J Mol Sci. 2020 Jul 16;21(14):5018. doi: 10.3390/ijms21145018 (PMC7404001; doi:10.3390/ijms21145018)
Supplement: Supplementary file 1 [file ijms-21-05018-s001.zip › Figure S5.pptx]

## Slide 1
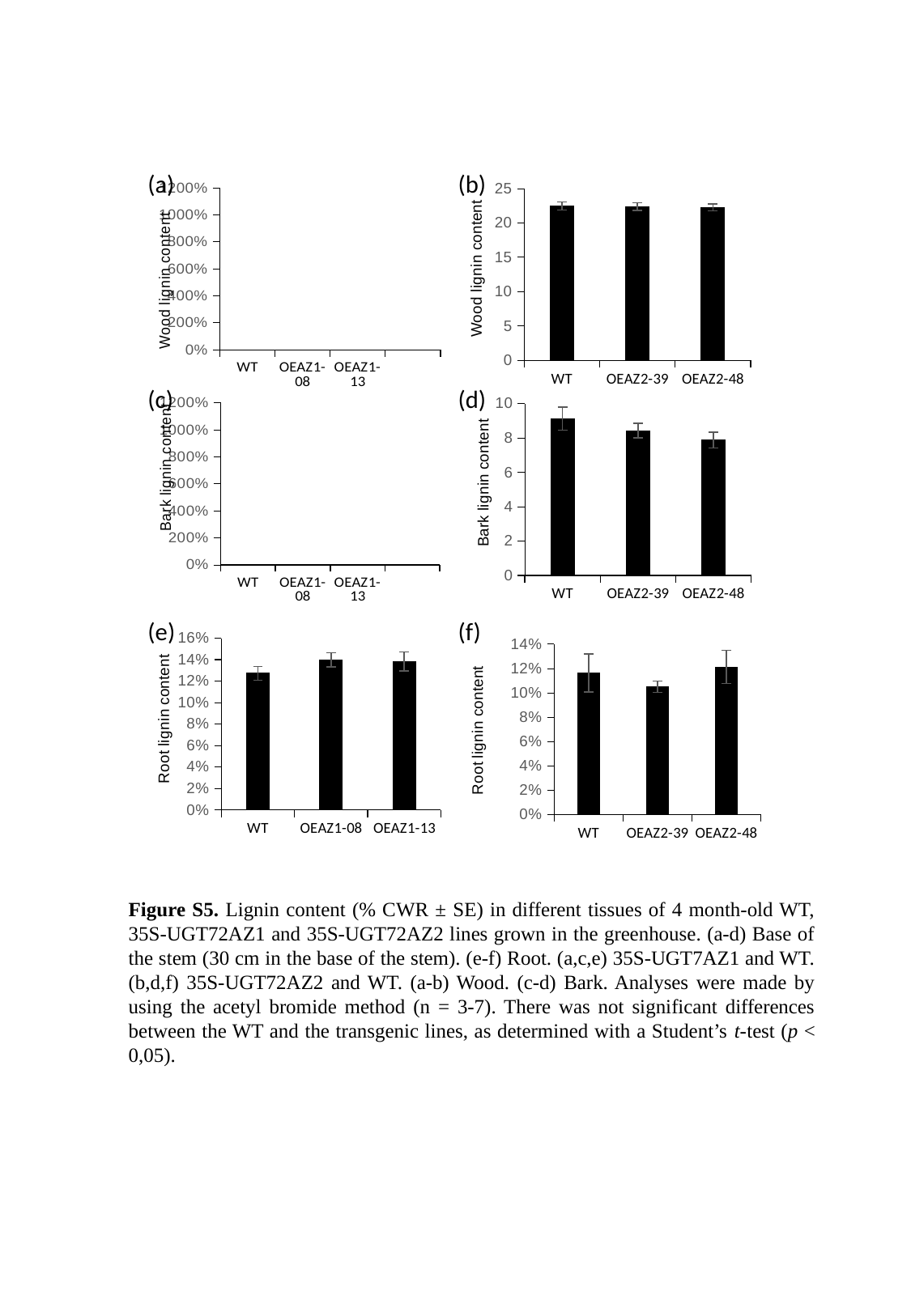

(a)
(b)
### Chart
| Category | moyenne |
|---|---|
| WT | 0.1294429945054945 |
| OEAZ1-08 | 0.12658516483516485 |
| OEAZ1-13 | 0.11110714285714285 |
### Chart
| Category | lignin (% CWR) |
|---|---|
| WT | 22.48815894177647 |
| OEAZ2-39 | 22.395062176931123 |
| OEAZ2-48 | 22.285062847838166 |(d)
(c)
### Chart
| Category | moyenne |
|---|---|
| WT | 0.09973351648351647 |
| OEAZ1-08 | 0.0906514423076923 |
| OEAZ1-13 | 0.0928021978021978 |
### Chart
| Category | lignin (% CWR) |
|---|---|
| WT | 9.135385665120669 |
| OEAZ2-39 | 8.434661397704245 |
| OEAZ2-48 | 7.886416475940405 |(e)
(f)
### Chart
| Category | moyenne |
|---|---|
| WT | 0.12727335164835163 |
| OEAZ1-08 | 0.139875 |
| OEAZ1-13 | 0.1383440934065934 |
### Chart
| Category | lignin (%CWR) |
|---|---|
| WT | 0.11650572424091588 |
| OEAZ2-39 | 0.10515679442508712 |
| OEAZ2-48 | 0.12130119009909951 |Figure S5. Lignin content (% CWR ± SE) in different tissues of 4 month-old WT, 35S-UGT72AZ1 and 35S-UGT72AZ2 lines grown in the greenhouse. (a-d) Base of the stem (30 cm in the base of the stem). (e-f) Root. (a,c,e) 35S-UGT7AZ1 and WT. (b,d,f) 35S-UGT72AZ2 and WT. (a-b) Wood. (c-d) Bark. Analyses were made by using the acetyl bromide method (n = 3-7). There was not significant differences between the WT and the transgenic lines, as determined with a Student’s t-test (p < 0,05).
