# Supplementary material for: Characterization of the UDP-glycosyltransferase UGT72 Family in Poplar and Identification of Genes Involved in the Glycosylation of Monolignols
Source: Int J Mol Sci. 2020 Jul 16;21(14):5018. doi: 10.3390/ijms21145018 (PMC7404001; doi:10.3390/ijms21145018)
Supplement: Supplementary file 1 [file ijms-21-05018-s001.zip › Figure S6.pptx]

## Slide 1
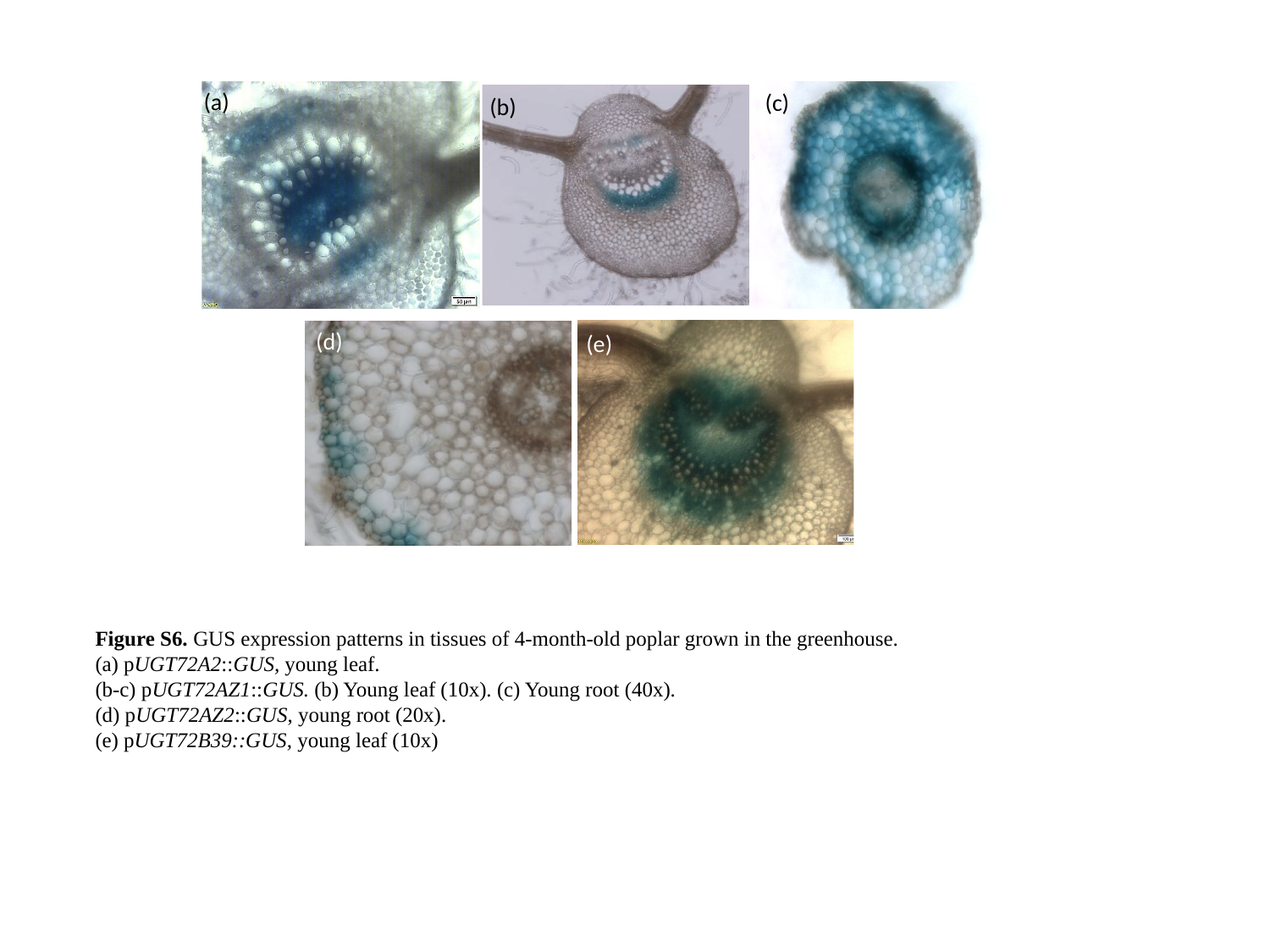

(a)
(c)
(b)
(d)
(e)
Figure S6. GUS expression patterns in tissues of 4-month-old poplar grown in the greenhouse.
(a) pUGT72A2::GUS, young leaf.
(b-c) pUGT72AZ1::GUS. (b) Young leaf (10x). (c) Young root (40x).
(d) pUGT72AZ2::GUS, young root (20x).
(e) pUGT72B39::GUS, young leaf (10x)
