# Supplementary material for: Characterization of the UDP-glycosyltransferase UGT72 Family in Poplar and Identification of Genes Involved in the Glycosylation of Monolignols
Source: Int J Mol Sci. 2020 Jul 16;21(14):5018. doi: 10.3390/ijms21145018 (PMC7404001; doi:10.3390/ijms21145018)
Supplement: Supplementary file 1 [file ijms-21-05018-s001.zip › Figure S7.pptx]

## Slide 1
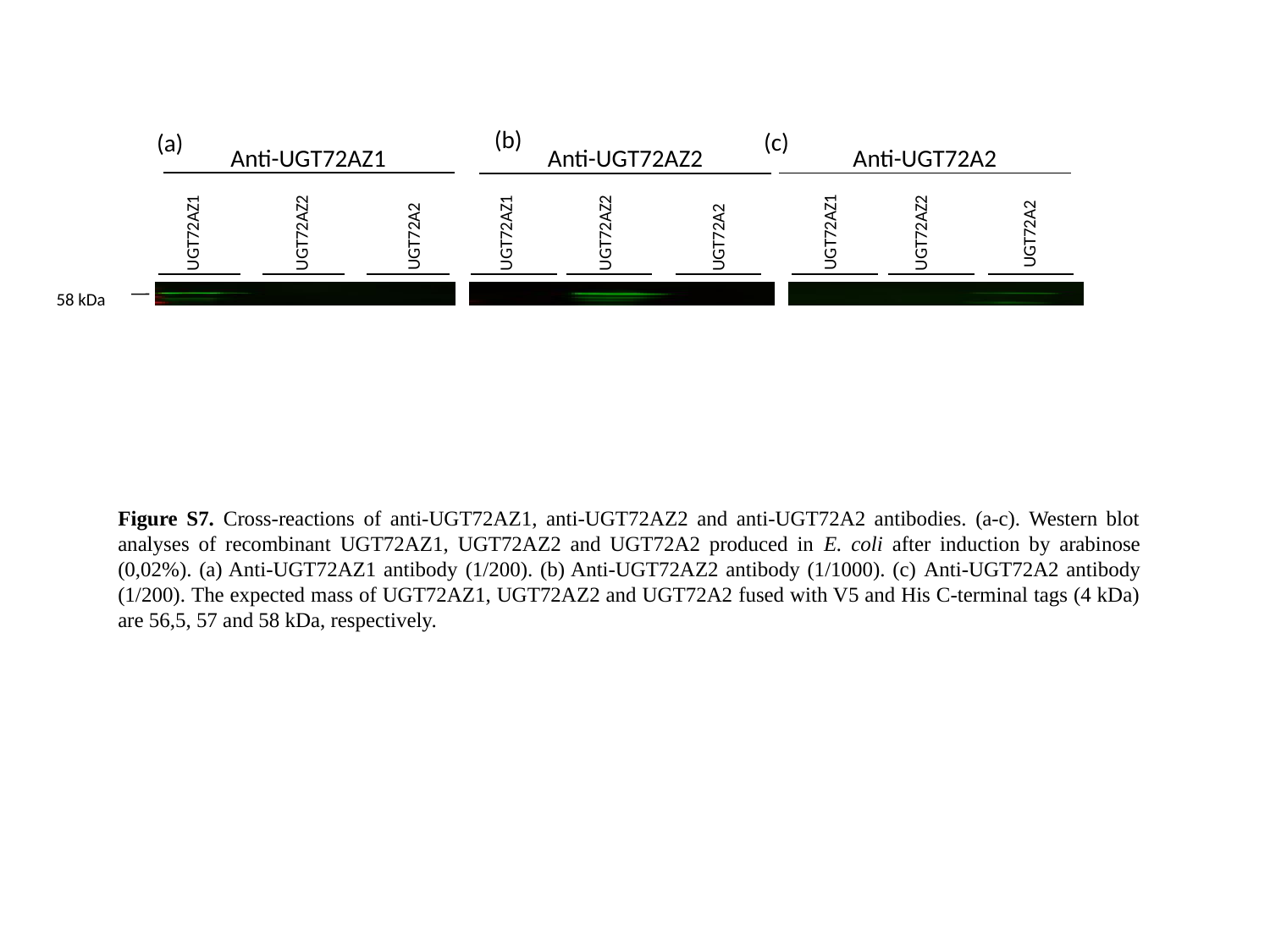

(b)
(c)
(a)
Anti-UGT72AZ1
Anti-UGT72AZ2
Anti-UGT72A2
UGT72AZ1
UGT72AZ1
UGT72AZ2
UGT72AZ1
UGT72AZ2
UGT72AZ2
UGT72A2
UGT72A2
UGT72A2
58 kDa
Figure S7. Cross-reactions of anti-UGT72AZ1, anti-UGT72AZ2 and anti-UGT72A2 antibodies. (a-c). Western blot analyses of recombinant UGT72AZ1, UGT72AZ2 and UGT72A2 produced in E. coli after induction by arabinose (0,02%). (a) Anti-UGT72AZ1 antibody (1/200). (b) Anti-UGT72AZ2 antibody (1/1000). (c) Anti-UGT72A2 antibody (1/200). The expected mass of UGT72AZ1, UGT72AZ2 and UGT72A2 fused with V5 and His C-terminal tags (4 kDa) are 56,5, 57 and 58 kDa, respectively.
